# Identifying links between cardiovascular disease and insomnia by modeling genes from a pleiotropic locus

**DOI:** 10.1242/dmm.052139

**Published:** 2025-05-06

**Authors:** Farah Abou Daya, Torrey Mandigo, Lily Ober, Dev Patel, Matthew Maher, Suraj Math, Cynthia Tchio, James A. Walker, Richa Saxena, Girish C. Melkani

**Affiliations:** ^1^ Department of Pathology, Division of Molecular and Cellular Pathology, Heersink School of Medicine, The University of Alabama at Birmingham, Birmingham, AL 35294, USA; ^2^ Center for Genomic Medicine, Massachusetts General Hospital, Boston, MA 02114, USA; ^3^ Department of Neurology, Massachusetts General Hospital, Harvard Medical School, Boston, MA 02114, USA; ^4^ Cancer Program, Broad Institute of MIT and Harvard, Cambridge, MA 02114, USA; ^5^ Department of Anesthesia, Critical Care and Pain Medicine, Massachusetts General Hospital and Harvard Medical School, Boston, MA 02114, USA; ^6^ UAB Nathan Shock Center, Birmingham, AL 35294, USA

**Keywords:** Cardiovascular disease, Insomnia, GWAS, Genetic loci, Inflammation, rs4643373 locus, *Drosophila* model

## Abstract

Insomnia symptoms double the risk of cardiovascular disease (CVD), yet shared genetic pathways remain unclear. Genome-wide association studies identified a genetic locus (near *ATP5G1*, *UBE2Z*, *SNF8*, *IGF2BP1* and *GIP*) linked to insomnia and CVD. We used *Drosophila* models to perform tissue-specific RNA interference knockdowns of four conserved orthologs (*ATPsynC*, *lsn*, *Bruce* and *Imp*) in neurons and the heart. Neuronal-specific knockdown of *ATPsynC*, *Imp* and *lsn* impaired sleep quantity and quality. In contrast, cardiac knockdown of *ATPsynC* and *lsn* reduced cardiac function and lifespan, with *lsn* knockdown also causing cardiac dilation and myofibrillar disorganization. Cross-tissue effects were evident: neuronal *Imp* knockdown compromised cardiac function, whereas cardiac *ATPsynC* and *lsn* knockdown increased sleep fragmentation and inflammation (marked by Upd3 elevation in the heart or head). Overexpression of Upd3 in neurons impaired cardiac function, and its overexpression in the heart disrupted sleep. Our findings reveal conserved genes mediating tissue-specific and cross-tissue interactions between sleep and cardiac function, providing novel insights into the genetic mechanisms linking insomnia and CVD through inflammation.

## Teaser

**Summary:** We knocked down cardiovascular disease- and insomnia-related genes in *Drosophila* neurons and heart, revealing impaired sleep, cardiac dysfunction and inflammation. Cross-tissue effects linked heart dysfunction to sleep disruption via Upd3.

## INTRODUCTION

Cardiovascular disease (CVD), one of the leading causes of death worldwide, encompasses several conditions that affect heart structure and function (Centers for Disease Control and Prevention, 2024; World Health Organization, 2024; Virani et al., 2021). The incidence of CVD continues to rise, with ∼18.2 million deaths worldwide in 2019, which contributes to rising healthcare costs and creates significant socioeconomic burden (Virani et al., 2021; Larsson and Markus, 2019). Factors that increase the risk of CVD include genetic factors, smoking and lack of physical activity. One important risk factor for CVD that has recently emerged is sleep dysfunction including insomnia (Larsson and Markus, 2019; Grandner et al., 2016; Zheng et al., 2019; Quan, 2009). Insomnia is the most common sleep disorder, affecting 10-30% of the population, and is defined as persistent difficulty in falling and/or staying asleep or non-restorative sleep resulting in daytime sleepiness, fatigue or dysfunction (Medline Plus; Sleep Foundation; Winkelman, 2015). Studies suggest that insomnia has a genetic component, with heritability estimates ranging between 22% and 25% in adults (Lind and Gehrman, 2016; Lane et al., 2019), and multiple genome-wide association studies (GWASs) have identified genetic loci with links to insomnia (Lane et al., 2019; Jansen et al., 2019; Song et al., 2020; Watanabe et al., 2020). Although genetic factors have been identified as contributors to CVD and insomnia, the genetic mechanisms underlying these two diseases remain poorly understood.

Observational studies have demonstrated that insomnia increases the risk of several disorders, especially CVD (Hoevenaar-Blom et al., 2011; Hsu et al., 2015; Javaheri and Redline, 2017; Bertisch et al., 2018). Moreover, Mendelian randomization analyses show that insomnia symptoms double the risk for incident CVD (Lane et al., 2019; Jansen et al., 2019). Similarly, cardiac dysfunction has been associated with sleep disruptions (Zheng, 2021; Parati et al., 2016). Although the mechanisms underlying these associations are poorly understood, a recent study found that sleep modifies atherosclerosis through hematopoiesis in mice (McAlpine et al., 2019). Together, these findings establish a clear link between cardiovascular traits and sleep disruptions. However, further insight into the specific underlying causal genetic pathways and mechanisms connecting CVD and insomnia is needed. Recent GWASs identified multiple significant loci for self-reported insomnia symptoms in UK Biobank and 23andMe participants (Lane et al., 2019; Jansen et al., 2019). From these loci, we identified a single locus, represented by a lead single-nucleotide polymorphism (SNP), rs4643373, that has also been previously associated with coronary artery disease (CAD) and other cardiac disorders, including myocardial infarction (van der Harst and Verweij, 2018; Khera and Kathiresan, 2017; Hartiala et al., 2021; Zhu et al., 2019; Said et al., 2022). This locus provides a valuable opportunity to identify genes important in CVD and/or insomnia and to dissect potential genetic mechanisms underlying the link between cardiovascular function and sleep. Near this locus, we identified five candidate genes: *ATP5G1* (also known as *ATP5MC1*), *UBE2Z*, *SNF8*, *IGF2BP1* and *GIP*. The known functions of these genes are diverse, including energy metabolism (*ATP5G1*), protein ubiquitination (*UBE2Z*), multivesicular body biogenesis (*SNF8*), post-transcriptional regulation (*IGF2BP1*) and lipid metabolism (*GIP*) (Saleh et al., 2022; Shi et al., 2020; Xu et al., 2017; Huang et al., 2018; Campbell and Drucker, 2013). However, it remains unclear which of these genes, if any, contribute to CVD or insomnia.

To elucidate the impact of these candidate genes on the regulation of cardiac function and sleep, we identified conserved *Drosophila melanogaster* orthologs for insomnia and CVD-related candidate genes within the locus*.* Whereas *ATPsynC* (*CG1746*), *Bruce* (*CG6303*), *lsn* (*CG6637*) and *Imp* (*CG1691*) were identified as *Drosophila* orthologs of *ATP5G1*, *UBE2Z*, *SNF8* and *IGF2BP1*, respectively, *GIP* lacks a *Drosophila* ortholog*. Drosophila* has become a well-established model system for studying both CVD and sleep disturbances (Piazza and Wessells, 2011; Bushey and Cirelli, 2011; Feng et al., 2018; Bhide et al., 2018; Gill et al., 2015; Wolf and Rockman, 2011). The fly heart displays many developmental and functional similarities to the mammalian heart (Piazza and Wessells, 2011; Souidi and Jagla, 2021). Moreover, several genes causing heart disease in humans are present in *Drosophila* and play similar pathophysiological roles, and the manipulation of these genes in *Drosophila* leads to disease phenotypes similar to those in humans (Piazza and Wessells, 2011; Souidi and Jagla, 2021; Wolf et al., 2006). Sleep in flies has also been demonstrated to share many characteristics with human sleep, such as consolidation during the night and similar responses to sleep-altering drugs (Bushey and Cirelli, 2011; Cirelli and Bushey, 2008; Beckwith and French, 2019; Nall and Sehgal, 2013). Therefore, studies investigating the role of human-relevant *Drosophila* orthologs in the regulation of cardiovascular function and sleep provide an efficient means to identify new causal genes related to CVD and/or insomnia and understand mechanisms relating both diseases to identify potential future therapeutic targets.

We hypothesized that genetic predisposition to CVD contributes to the development of insomnia, and vice versa, through shared mechanisms. To address this, we evaluated the cardiac- and sleep-specific roles of *Drosophila* genes *ATPsynC*, *Bruce*, *lsn* and *Imp* in both cell-autonomous and non-cell-autonomous manners*.* To assess the role of these genes in cardiac and sleep physiology, we performed tissue-specific knockdown (KD) in the heart and nervous system, respectively. Cardiac- and neuronal-specific KD of these genes led to cardiac and sleep dysfunction, suggesting tissue-specific functions related to each disease. After characterizing the cell-autonomous role of *ATPsynC*, *Bruce*, *lsn* and *Imp* in cardiac function and sleep, we also identified non-cell-autonomous effects of these genes on cardiac phenotypes when knocked down neuronally and sleep phenotypes when knocked down in cardiac tissue. Neuronal KD of *Imp* compromised cardiac function, whereas cardiac KD of *ATPsynC*, *lsn* and *Bruce* led to sleep dysfunction in a non-cell-autonomous manner. We further identified inflammation as an underlying mechanism involved in the non-cell-autonomous effects of cardiac dysfunction on sleep disruption and vice versa. In conclusion, we were able to uncover novel genetic mechanisms with cell-autonomous effects on the regulation of cardiac function and sleep, as well as non-cell autonomous genetic mechanisms linking cardiac function in the regulation of sleep and effects of sleep on cardiac function. Taken together, our data are among the first functional genetic evidence linking CVD with sleep disorders and provide mechanistic insight into potential therapeutic targets to prevent or attenuate both diseases.

## RESULTS

### A shared GWAS locus at 17q21 is associated with CAD/CVD and insomnia

**Fig. 1.**
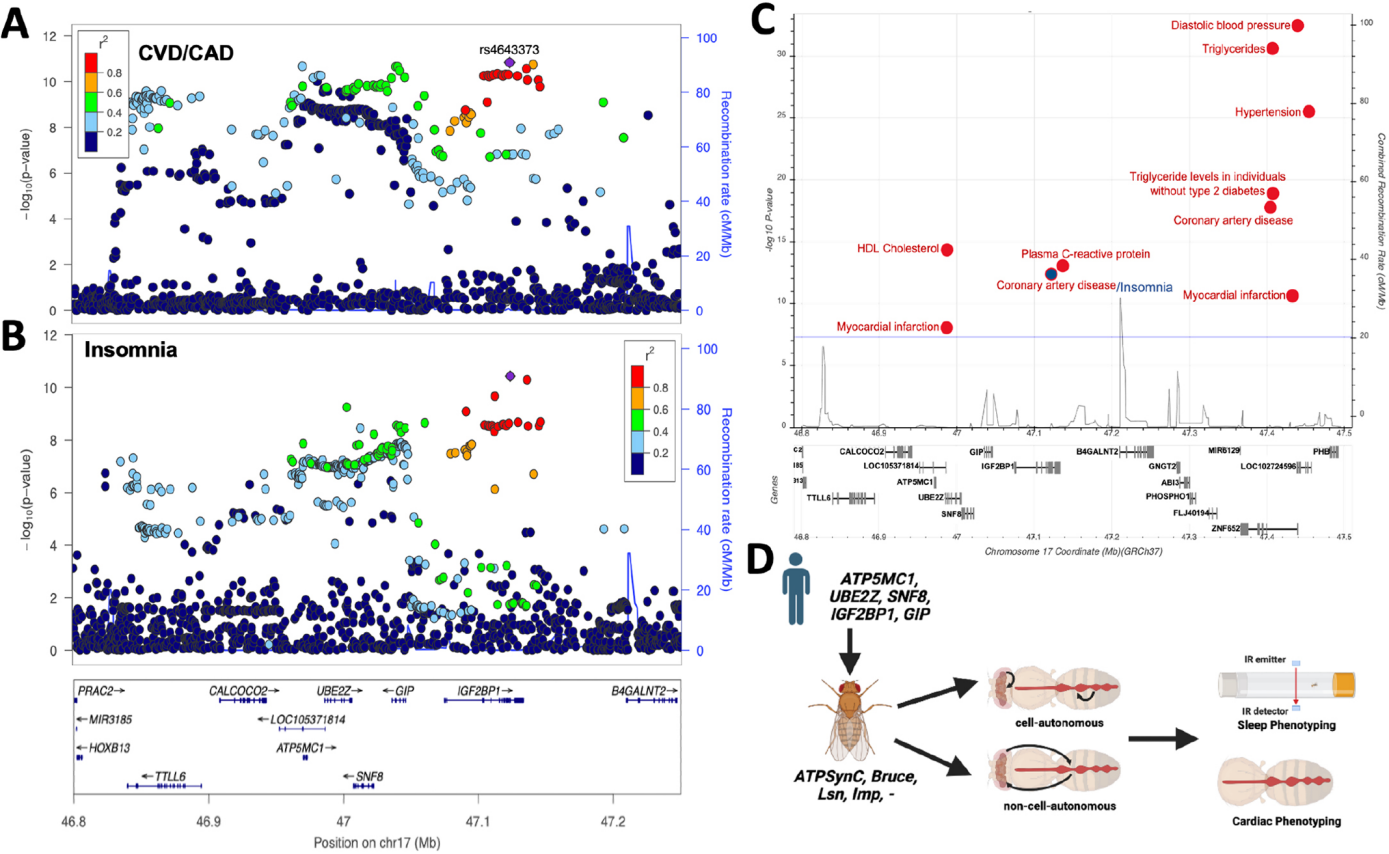
**The cardiovascular disease (CVD)- and insomnia-related locus, other phenotypic associations and nearby genes.** (A,B) Manhattan plots (LocusZoom) showing CVD (A) and insomnia (B) single-nucleotide polymorphism association peaks with five nearby candidate genes: *ATP5MC1* (*ATP5G1*), *UBE2Z*, *SNF8*, *GIP* and *IGF2BP1*. (C) Genome-wide association study (GWAS) associations meeting genome-wide significance (*P*<^5×10−8^; blue line) for cardiovascular/cardiometabolic traits in the region near rs4643373 obtained from the Cardiovascular Disease Knowledge Portal (see Materials and Methods) and literature (Watanabe et al., 2022). Each point corresponds to a single trait in a single study. The blue dot represents rs4643373. (D) Graphical scheme showing the experimental workflow. IR, infra-red. Created in BioRender by Abou Daya, F. (2025). https://BioRender.com/65wcnj5. This figure was sublicensed under CC-BY 4.0 terms.

A GWAS signal at 17q21.32 for CAD including myocardial infarction, percutaneous transluminal coronary angioplasty, coronary artery bypass grafting, angina or chronic ischemic heart disease from the CARDIoGRAMplusC4D Consortium, represented by the lead SNP rs4643373 [Fig. 1A, *n*=181,522 cases and 984,079 controls; odds ratio (OR)=1.04 (95% c.i.=1.028-1.050)] (Aragam et al., 2022) was found to colocalize with an association signal for insomnia symptoms [*n*=593,724 cases and 1,771,286 controls; OR=1.04 (95% c.i.=1.025-1.046); Fig. 1B; posterior probability (pp)=0.95 that both traits share the same causal SNP] (Watanabe et al., 2022). Notably, the colocalized signal is located within the intronic region of *IGF2BP1*, and SNPs in linkage disequilibrium with this signal are found to overlap with four other genes, *ATP5G1*, *UBE2Z*, *SNF8* and *GIP* (Fig. 1A,B). Moreover, multi-tissue expression quantitative trait locus (eQTL) analyses show an effect of lead SNP rs4643373 on the expression of these genes in multiple tissues, including the left ventricle of the heart and the hypothalamus (Table S1). This genomic context provided the rationale for further investigating these four genes to understand the potential biological significance of this signal in the context of CVD and insomnia. Furthermore, to check for other CVD-related signals in the region of rs4643373, we catalogued cardiovascular and cardiometabolic trait associations at genome-wide significance (*P*<5×10^−8^) of this region from literature (Watanabe et al., 2022) and the Cardiovascular Disease Knowledge Portal (see Materials and Methods). We found a total of nine significant associations with cardiovascular and cardiometabolic traits, including myocardial infarction (Fig. 1C), reinforcing the importance of this genomic region in CVD. The causal genes and variants at this locus are unknown. Furthermore, it is unclear whether the association signals reflect independent contribution of effector genes to sleep and CVD, or whether effector genes influence sleep through cardiovascular dysfunction or vice versa. Thus, we set out to identify the role of *Drosophila* orthologs of these genes, detailed in Table 1, in sleep and cardiovascular function in both cell-autonomous and non-cell-autonomous manners (Fig. 1D).

**Table 1.** Human and fly cardiovascular disease- and insomnia-related genes, with percentage similarity at a single locus as determined by DIOPT (Hu et al., 2021)

Human symbol	*Drosophila* symbol	Percentage similarity
*ATP5G1*	*ATPsynC*	83
*UBE2Z*	*Bruce*	54
*SNF8*	*lsn*	71
*IGF2BP1*	*Imp*	58
*GIP*	−	−

### Neuronal-specific suppression of CVD- and insomnia-related genes leads to altered sleep phenotypes along with sleep fragmentation

To assess whether any of the four orthologs of the genes within the CVD- and insomnia-associated locus (Table 1) were essential in *Drosophila*, we performed ubiquitous KD driven by the *Act5C-Gal4* driver*.* Ubiquitous RNA interference (RNAi) KD of *ATPsynC* and *lsn* led to lethality, whereas that of *Bruce* and *Imp* did not affect viability. We also tested viability with pan-neuronal KD using the *elav-Gal4* driver. Only *ATPsynC* Line 1 (see Materials and Methods) led to lethality when suppressed pan-neuronally, suggesting an essential role for *ATPsynC* in neuronal function.

**Fig. 2.**
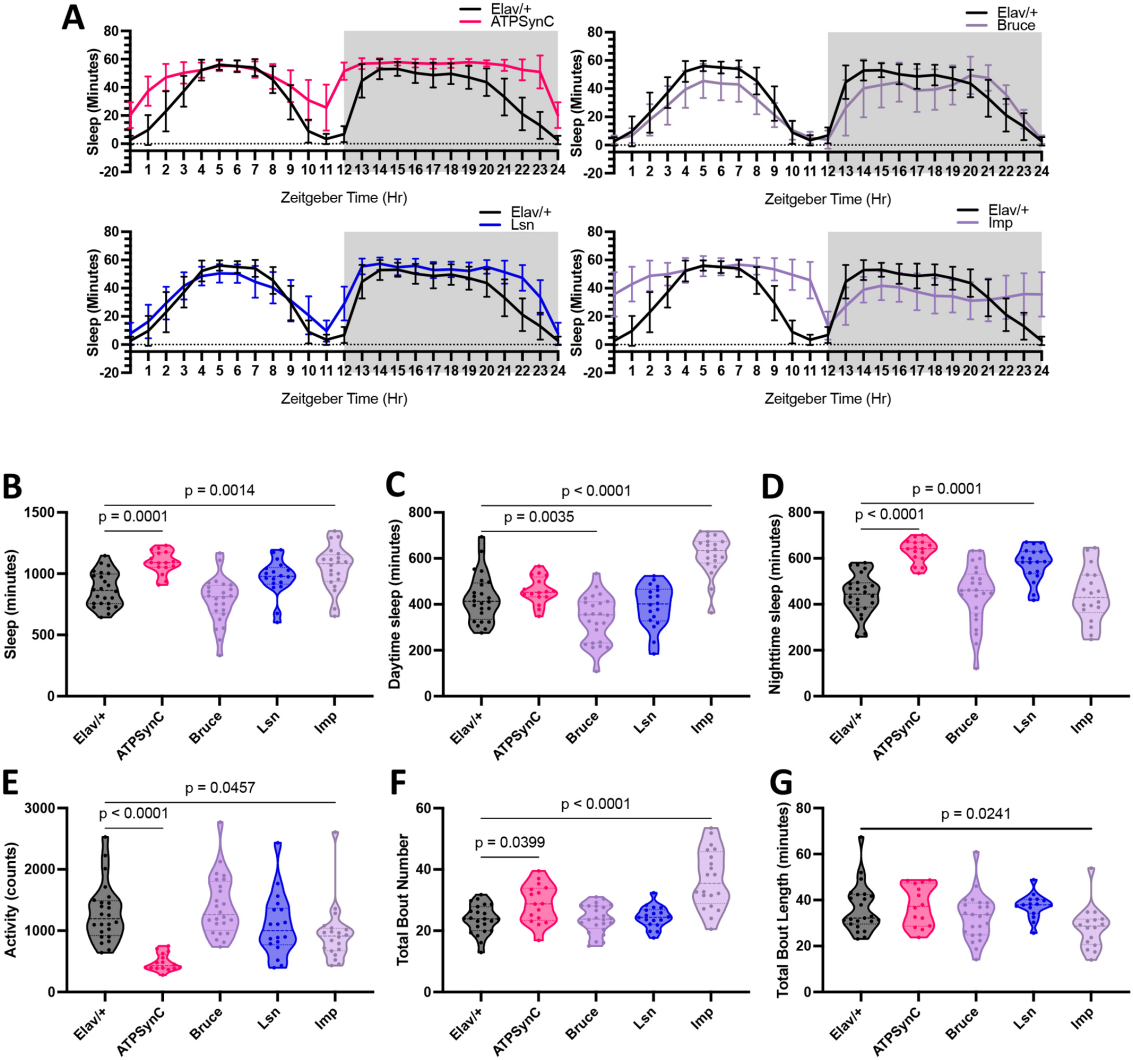
**Neuronal-specific suppression of CVD- and insomnia-related genes leads to compromised sleep phenotypes.** (A) Sleep profiles showing sleep minutes per hour for 24 h. (B-G) Violin plots for quantitative sleep parameters – total sleep amount (B), daytime sleep amount (C), nighttime sleep amount (D), total locomotor activity (E), total bout number (F) and total bout length (G) – in 1-week-old male *Drosophila* with neuronal RNA interference (RNAi) knockdown of CVD- and insomnia-related genes with the pan-neuronal *elav-Gal4* driver. *n*=16-24 for each group. For *ATPsynC*, Line 1 was lethal; thus, Line 2 was used (refer to Materials and Methods). Data were collected from at least two independent experiments from one RNAi line (second line data shown in Fig. S2). Each data point represents a fly. Statistics were calculated by one-way ANOVA for comparisons to controls.

To test the impact of these four genes near the CVD- and insomnia-associated locus on sleep, we used the neuronal-specific *elav-Gal4* driver to knock down gene expression using RNAi. The level of KD of each gene in the head is shown in Fig. S1A-D. Because *elav-Gal4* KD of *ATPsynC* using RNAi Line 1 caused lethality, we used another available line that causes efficient KD using the same driver (Fig. S1A; see Materials and Methods). Compared to driver (Fig. 2) and UAS control (Table S2) flies, RNAi-mediated inhibition of *ATPsynC* significantly increased overall sleep duration, primarily from an increase in nighttime sleep (Fig. 2A-D). This increased sleep corresponded with a decrease in overall locomotor activity (Fig. 2E). KD of *Imp* also resulted in increased sleep, primarily through increased daytime sleep (Fig. 2A-D) and decreased activity (Fig. 2E). Even though the suppression of *lsn* resulted in a significant increase in nighttime sleep (Fig. 2A-D), it was not significantly different from that in its UAS control. However, neuronal KD of *Bruce* decreased daytime sleep but did not affect overall sleep or activity (Fig. 2A-E). Although we observed differences in daytime or nighttime sleep with different RNAi lines for *lsn* or *Imp* flies, respectively, both lines for each gene affected total sleep in a consistent manner. These differences could be attributed to differences in RNAi efficiencies between each line. Moreover, we observed similar sleep and activity trends in *ATPsynC*, *lsn* and *Bruce* females, but only a decrease in nighttime sleep in *Imp* females compared to controls without changes in other parameters (Fig. S3). Because we observed a decrease in activity accompanying an increase in sleep, we evaluated fly locomotion speed using Massively Automated Real-time GUI for Object-tracking (MARGO) (Werkhoven et al., 2019; Elya et al., 2023). For both *ATPsynC* and *Imp* males, we observed lethargic flies with a decrease in locomotion speed (Fig. S4A). However, *ATPsynC* females, which also showed an increase in sleep, did not have significantly affected locomotion speed (Fig. S4B). This suggests that the increase in sleep in *ATPsynC* flies is a true increase in sleep and not caused by slower-moving flies falsely increasing the time between beam breaks in the *Drosophila* activity monitor (DAM) system. However, this is not the case for the longer sleep observed in male *Imp* flies, as the increased sleep only occurs when their locomotion speeds are reduced, as demonstrated by the comparison between male and female *Imp* flies.

To further characterize these sleep alterations observed upon KD of each gene (summarized in Table S4), we measured the number and length of sleep bouts to assess sleep quality. Compared to driver and UAS controls, KD of only *Imp* resulted in increased sleep bout number along with decreased bout length, indicative of sleep fragmentation (Fig. 2F,G). We also observed similar sleep and activity trends in 3-week-old male flies (Fig. S5). Moreover, to assess the effects of adult-specific KD of those genes, we used the GeneSwitch system and observed an increased sleep phenotype in *ATPsynC* flies, similar to that observed when the gene was knocked down throughout development, indicating a minimal role for *ATPsynC* during development toward the regulation of sleep (Fig. S6). Conversely, sleep dysfunction observed upon *Imp* KD was lost with adult-specific KD of this gene, indicating a developmental role of *Imp* in the regulation of sleep (Fig. S6). To determine the effect of KD of these genes in regulating circadian rhythms, we observed flies under constant darkness. We found that neuronal-specific KD of *Imp* led to loss of rhythmicity in all flies tested, indicating disrupted circadian rhythms (Table S3). KD of *ATPsynC* decreased the number of rhythmic flies (41.18%) but did not significantly affect the period length or rhythm strength, whereas KD of *lsn* or *Bruce* did not result in circadian disruptions (Table S3). Therefore, the neuronal suppression of genes within the CVD- and insomnia-related locus led to a significantly altered sleep phenotype characterized by an increase in overall sleep for two genes (*ATPsynC* and *Imp*), with only *Imp* affecting sleep fragmentation and circadian rhythms.

### Cardiac-specific suppression of CVD- and insomnia-related genes leads to cardiac dysfunction, myofibrillar disorganization, cardiac fibrosis and shortened lifespan

**Fig. 3.**
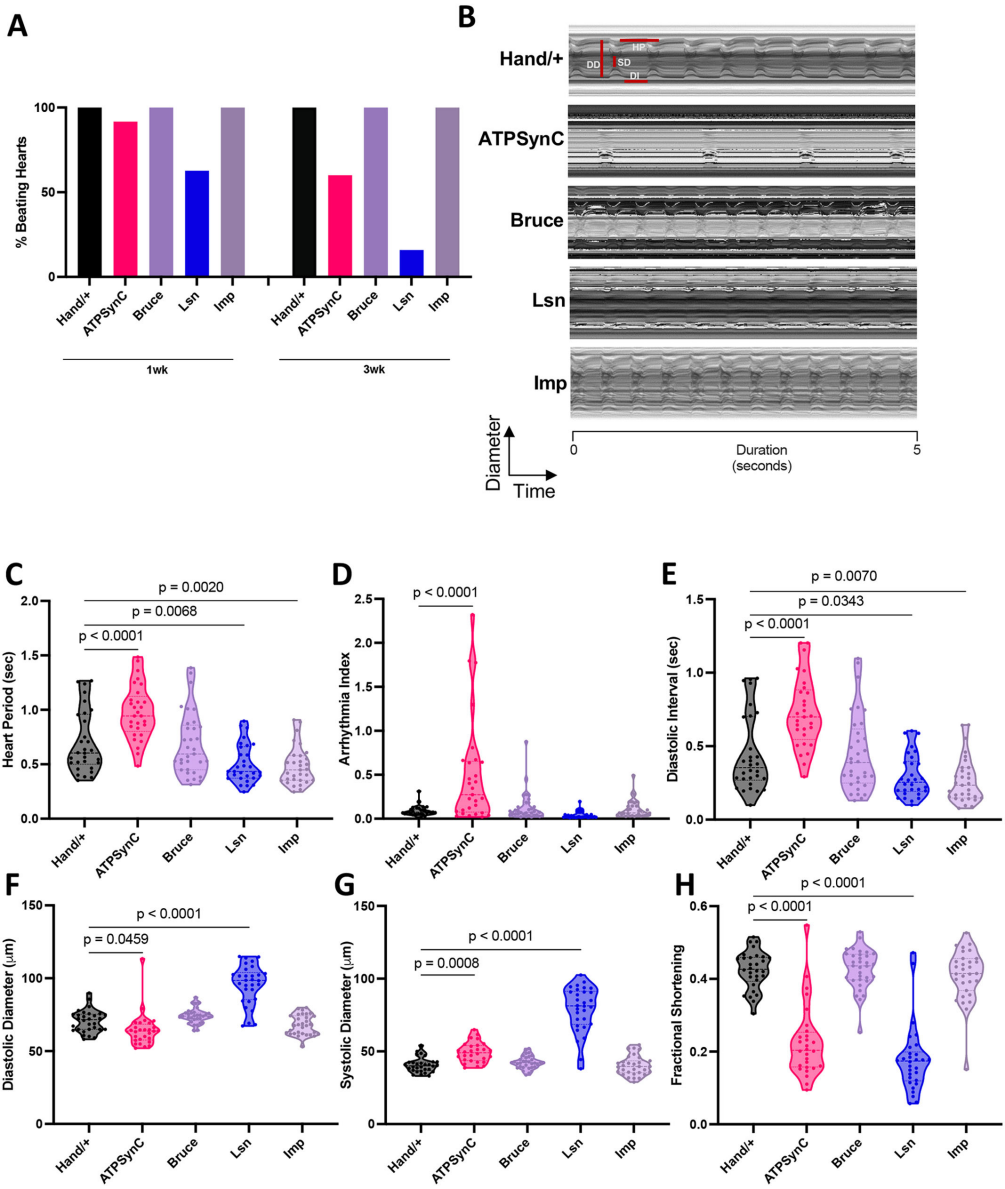
**Cardiac-specific suppression of CVD- and insomnia-related genes leads to cardiac dysfunction.** (A) The percentage of beating hearts at 1 versus 3 weeks of age shows a significant effect of *lsn* knockdown with age (*P*<0.0001). (B) Representative 5-s mechanical modes from 1-week-old male flies with cardiac RNAi knockdown of CVD- and insomnia-related genes with cardiac-specific *Hand-Gal4* driver. DD, diastolic diameter; DI, diastolic interval; HP, heart period; SD, systolic diameter. (C-H) Violin plots for cardiac physiological parameters – heart period (C), arrythmia index (D), diastolic interval (E), diastolic diameter (F), systolic diameter (G) and fractional shortening (H) – in 1-week-old male flies. *n*=29-33 for each group for C-H; *n*=32-51 for each group for A. Each data point represents one fly. Data were collected from at least two independent experiments from one RNAi line (second line data shown in Fig. S2). Statistics were calculated by one-way ANOVA for C-H; Fisher's exact test was performed for A.

To assess whether the function of these genes was essential in all muscle tissues, we performed a pan-muscle KD using the *24b-Gal4* driver. As with ubiquitous KD, we found that pan-muscle KD of *ATPsynC* and *lsn* resulted in lethality, whereas flies with pan-muscle KD of *Bruce* and *Imp* were viable. Next, to evaluate the effect of suppressing these genes on cardiac performance, KD of *ATPsynC*, *Bruce*, *lsn* or *Imp* was carried out using the cardiac-specific *Hand-Gal4* driver. Levels of KD of each gene in the heart are shown in Fig. S7A-D. One-week-old male and female flies were dissected and imaged for assessment of cardiac physiological parameters. Most strikingly, suppressing *lsn* led to a non-beating, heart failure-like phenotype where only 62.75% of hearts beat at 1 week of age, which decreased to 15.91% by 3 weeks of age in males (Fig. 3A). *ATPsynC* flies also exhibited a decrease in the number of beating hearts with age, from 91.67% at 1 week to 60% at 3 weeks of age (Fig. 3A).

We then analyzed cardiac physiological parameters including heart period (HP), which refers to the time interval between successive heartbeats and serves as an important measure of heart rate; arrhythmia index (AI), which quantifies the degree of irregularity or arrhythmic events in the heart rate, which can indicate disruptions in electrical conduction; and diastolic interval (DI), which measures the duration of diastole, the phase in which the heart relaxes and fills with blood, which informs on the efficiency of the heart's filling capacity. We also measured diastolic diameter (DD), which refers to the diameter of the heart at the end of diastole, reflecting cardiac chamber size; systolic diameter (SD), which measures the size of the heart at the peak of contraction, reflecting the heart's ability to contract and pump blood effectively; and fractional shortening (FS), which assesses the percentage change in the heart's diameter from diastole to systole, providing a direct measure of cardiac contractility and performance. We summarized the results in Table S4. Upon analyzing beating hearts, and in comparison to driver (Fig. 3) and UAS controls (Table S2), cardiac-specific KD of *ATPsynC* resulted in a bradycardic phenotype with cardiac dysfunction characterized by significantly increased HP, AI, DI and SD, and reduced DD and FS, a measure of cardiac performance, in both male and female flies (Fig. 3B-H; Fig. S8A). Suppression of *lsn* led to cardiac dysfunction characterized by significantly increased DD and SD, and reduced FS in both sexes, in addition to a tachycardic phenotype in males characterized by decreased HP (Fig. 3B-H; Fig. S8A). Suppression of *Imp* led to tachycardia characterized by decreased HP without affecting cardiac function in males only (Fig. 3B-H; Fig. S8A). Suppressing *Bruce* in males and females did not significantly affect heart function in 1-week-old flies (Fig. 3B-H; Fig. S8A). To assess whether the effects of these genes on the heart persist with age, we assessed cardiac function in 3-week-old male flies with cardiac-specific suppression of each gene and observed similar overall trends for cardiac parameters as observed in 1-week-old *ATPsynC* and *lsn* flies (Fig. S9). Interestingly, in 3-week-old flies with KD of *Bruce*, we observed cardiac dysfunction, which was not present at 1 week of age, characterized by increased DD and SD, and decreased FS (Fig. S9), suggesting an age-related component involved in the cardiac phenotype observed with *Bruce* KD. To determine the effect of adult-specific KD of these genes, we used the GeneSwitch system again. Upon adult-specific KD of *ATPsynC* or *lsn*, we observed significantly decreased FS, similar to that observed upon KD throughout development, thus suggesting that these defects in cardiac function are not developmental and rather arise from the disruption of mechanisms active during adulthood (Fig. S10).

**Fig. 4.**
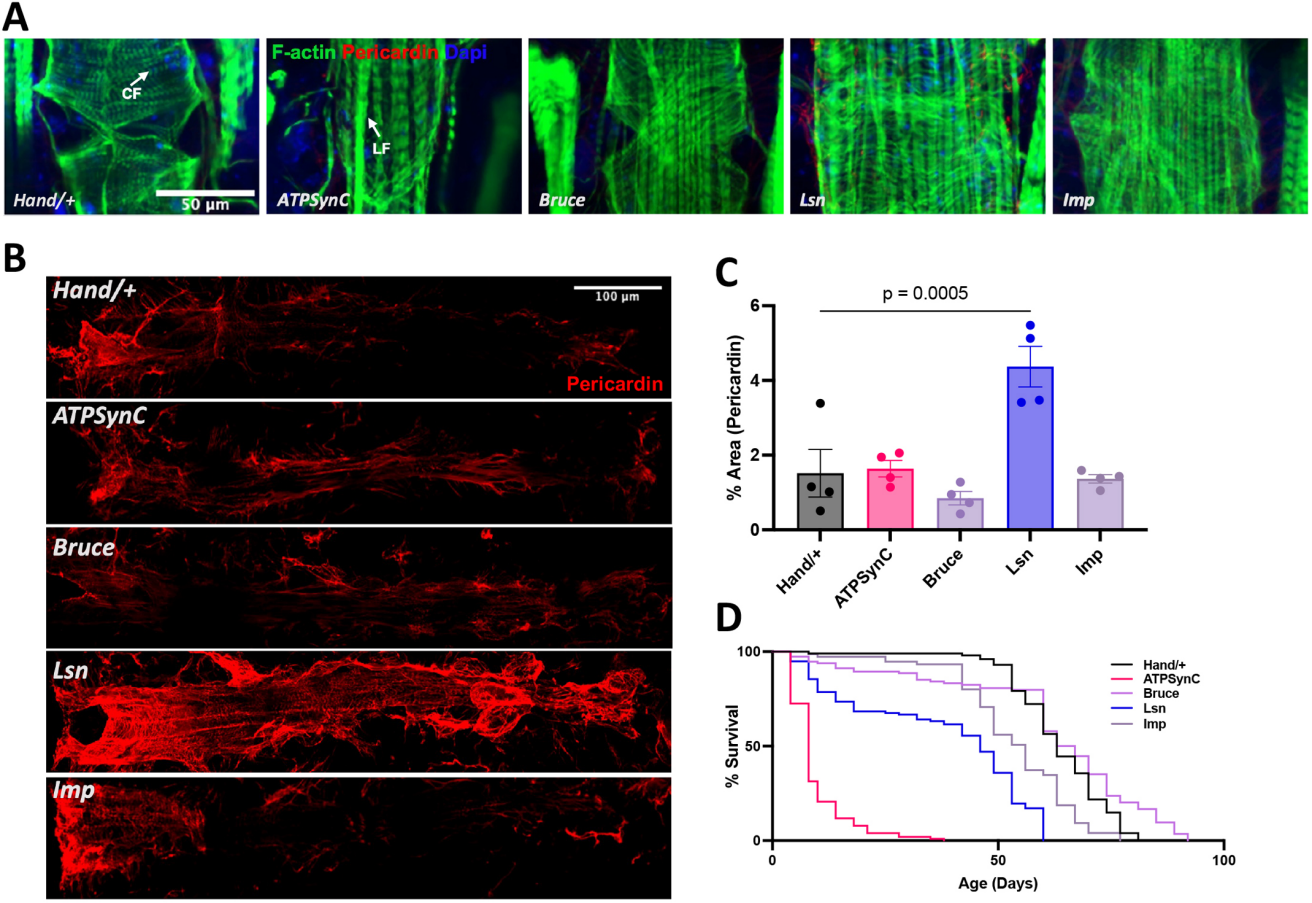
**Cardiac-specific suppression of CVD- and insomnia-related genes leads to myofibrillar disorganization, cardiac fibrosis and shortened lifespan.** (A,B) Representative images showing Actin-containing myofibrils (A) and Pericardin (B) in 1-week-old male flies with cardiac RNAi knockdown of CVD- and insomnia-related genes with *Hand-Gal4*. (C) Quantification of Pericardin signal. Each data point is for a single fly. (D) Lifespan assay for male flies with cardiac RNAi knockdown of CVD- and insomnia-related genes with cardiac-specific *Hand-Gal4* driver resulted in significant decrease in lifespan (*P*<0.0001) of *ATPsynC*, *lsn* and *Imp* flies, and a significant increase in lifespan of *Bruce* flies (*P*=0.0057). Graph plots percentage survival (*n*>100 for each group) versus time post-eclosion. Statistics were calculated by one-way ANOVA for C; a Kaplan–Meier test was performed for D.

To better understand what underlies these cardiac defects, we characterized the morphology of hearts with KD of each gene by staining 1-week-old male hearts with phalloidin. KD of *ATPsynC* severely disrupted Actin-containing myofibrillar organization and led to almost complete loss of contractile circumferential muscles (CF) and mostly non-contractile longitudinal muscles (LF) being seen (Fig. 4A; Fig. S11A). KD of *lsn* resulted in a dilated heart with more evident CF aggregations along with myofibrillar disarray, whereas that of *Bruce* and *Imp* resulted in a less severe phenotype, with visible CFs and LFs (Fig. 4A; Fig. S11A). Moreover, only suppression of *lsn* led to significantly increased Pericardin deposition, which is a collagen-like protein and a component of the extracellular matrix, indicative of a fibrotic phenotype (Fig. 4B,C). We also assessed the effects of cardiac KD of these genes on lifespan. Cardiac suppression of *ATPsynC*, *lsn* and *Imp* led to a significantly shortened lifespan in both sexes (*P*<0.0001), whereas suppression of *Bruce* resulted in an increased lifespan in males (*P*=0.0057) (Fig. 4D; Fig. S8B). Our findings indicate that suppression of CVD- and insomnia-related genes *lsn* and *ATPsynC* in the heart led to significantly compromised cardiac function with myofibril disorganization and shortened lifespan. Moreover, suppression of *Bruce* and *Imp* resulted in less severe phenotypes, with *Bruce* KD leading to cardiac dysfunction with age.

### Non-cell-autonomous mechanisms linking CVD with insomnia

**Fig. 5.**
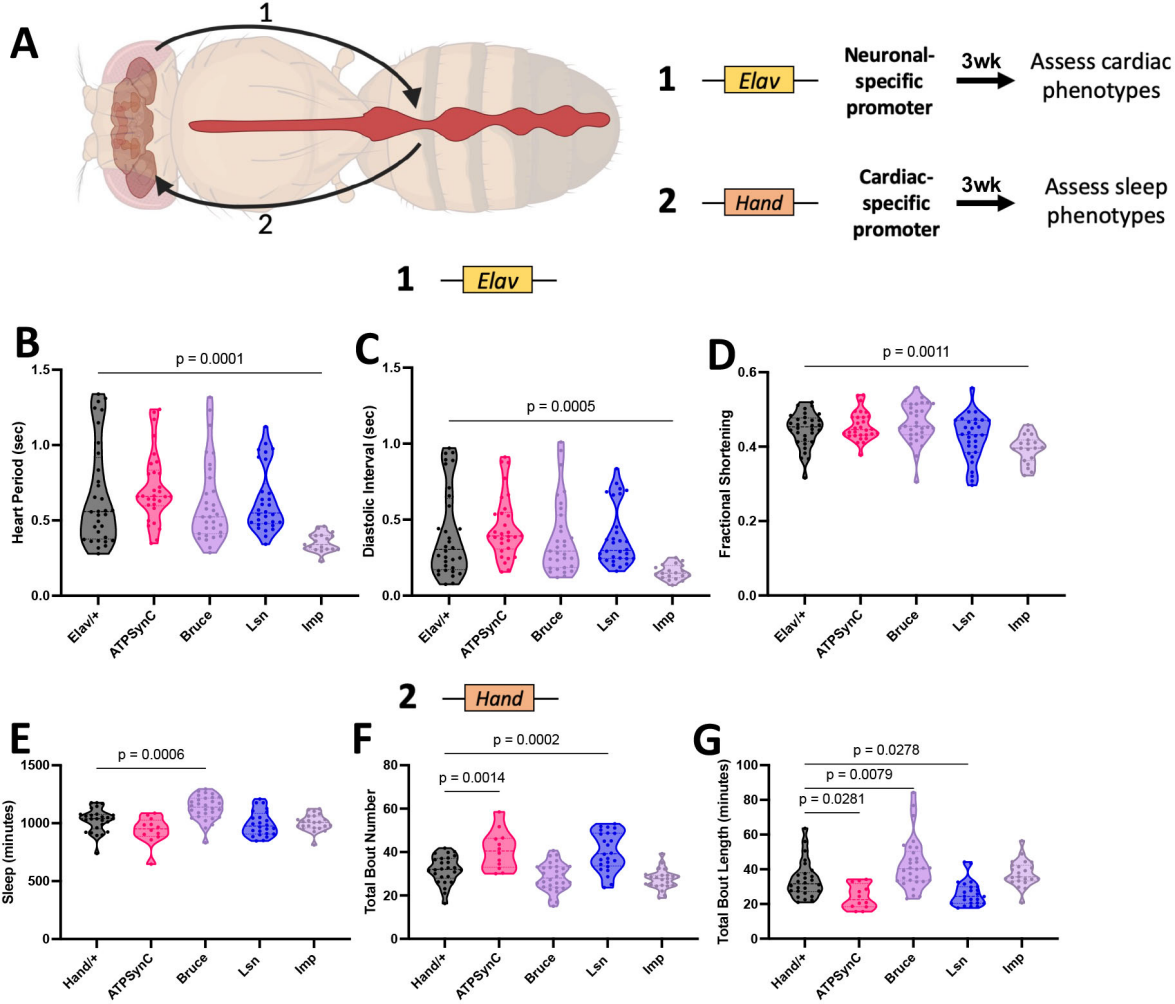
**Non-cell-autonomous mechanisms linking CVD with insomnia.** (A) Graphical scheme showing the experimental layout. Created in BioRender by Abou Daya, F. (2025). https://BioRender.com/5pgdyfi. This figure was sublicensed under CC-BY 4.0 terms. (B-D) Violin plots for cardiac physiological parameters – heart period (B), diastolic interval (C) and fractional shortening (D) – in 3-week-old male flies with neuronal-specific knockdown of CVD- and insomnia-related genes (*n*=19-32 per group). (E-G) Violin plots for quantitative sleep parameters – total sleep amount (E), total bout number (F) and total bout length (G) – in 3-week-old male flies with cardiac-specific knockdown of CVD- and insomnia-related genes. *n*=12-30 per group. Each data point represents one fly. Statistics were calculated by one-way ANOVA.

Mendelian randomization analysis has confirmed a causal role for insomnia in CVD (Lane et al., 2019). Moreover, cardiac dysfunction has also been associated with sleep disruptions (Zheng, 2021; Parati et al., 2016; Sharma et al., 2010). Therefore, to assess the non-cell-autonomous roles of these genes in influencing cardiac and sleep dysfunction, we suppressed gene expression in neurons and measured cardiac function, or we suppressed expression in the heart and assessed sleep phenotypes (Fig. 5A). Both heart and sleep phenotyping were conducted in 3-week-old male flies to allow for cardiac dysfunction or sleep dysfunction to accumulate and increase the likelihood of non-cell-autonomous effects. When assessing the non-cell-autonomous effects of pan-neuronal KD on cardiac function, we found that, unlike cardiac-specific KD, neuronal suppression of *ATPsynC*, *lsn* or *Bruce* resulted in no cardiac phenotype. However, neuronal suppression of *Imp* in 3-week-old male flies, which cell-autonomously increased sleep fragmentation (Fig. 2), significantly decreased HP and DI, and significantly reduced FS (Fig. 5B-D). Together, these data suggest a non-cell-autonomous role for *Imp*, in neurons, in regulating cardiac performance through sleep disruption characterized by increased sleep fragmentation.

When assessing the non-cell-autonomous effects of cardiac KD on sleep, we found that only cardiac-specific KD of *Bruce* in 3-week-old flies affected overall sleep quantity, leading to a significant increase in total sleep (Fig. 5E). We then assessed sleep quality by measuring the sleep structure of flies with cardiac KD of each gene, by evaluating the total number of sleep bouts and the average length of those sleep bouts. Only cardiac KD of *ATPsynC* and *lsn* resulted in significant increases in sleep bouts that were significantly shorter in length (Fig. 5F,G), indicative of more fragmented sleep. Conversely, cardiac KD of Bruce resulted in significantly longer sleep bouts compared to those of controls, with no change in the amount of sleep bouts (Fig. 5F,G), leading to the increase in overall sleep observed. Together, these data demonstrate a non-cell-autonomous effect of cardiac dysfunction on sleep.

**Fig. 6.**
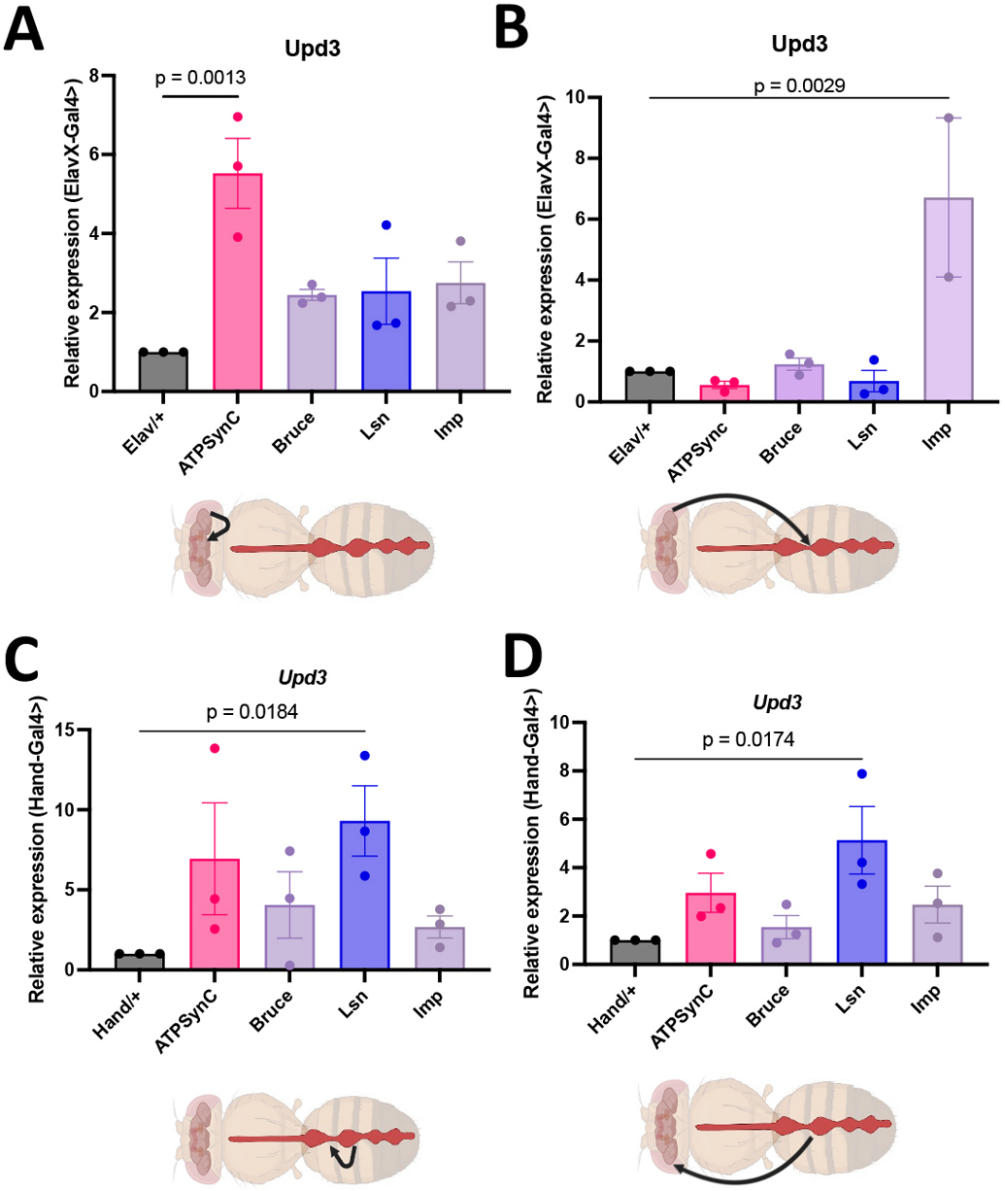
**Knockdown of CVD- and insomnia-related genes increases inflammation cell-autonomously and non-cell-autonomously.** (A) Transcript levels of *upd3* in heads of flies with neuronal-specific knockdown of the indicated genes (*n*=10-12 heads per data point per group). (B) Transcript levels of *upd3* in hearts of flies with neuronal-specific knockdown of the indicated genes (*n*=10-12 hearts per data point per group). (C) Transcript levels of *upd3* in hearts of flies with cardiac-specific knockdown of the indicated genes (*n*=10-12 hearts per data point per group). (D) Transcript levels of *upd3* in heads of flies with cardiac-specific knockdown of the indicated genes (*n*=10-12 heads per data point per group). Statistics were calculated by one-way ANOVA. Created in BioRender by BioRender. Abou Daya, F. (2025). https://BioRender.com/6kvmu5y. This figure was sublicensed under CC-BY 4.0 terms.

Because inflammation has been suggested as a potential mechanism underlying the connection between CVD and sleep dysfunction (Javaheri and Redline, 2017), we measured levels of an *IL6*-like proinflammatory cytokine (Romão et al., 2021), *upd3*, in the heads and hearts of flies with either neuronal-specific KD (Fig. 6A,B) or cardiac-specific KD (Fig. 6C,D). Although neuronal KD of all genes tested led to increased levels of *upd3* in the head, only *ATPsynC* KD led to a significant increase in *upd3* expression (Fig. 6A). Moreover, neuronal KD of *Imp* significantly increased *upd3* expression in the heart (Fig. 6B). Overall, these data, along with the effects of neuronal *Imp* KD on sleep and cardiac function, suggest that *upd3* expression mediates the non-cell-autonomous effects of *Imp* on the heart through an increased inflammatory state.

We then measured the levels of *upd3* in the heart upon cardiac-specific KD of each gene. Although KD of *Bruce* and *Imp* had minimal effects on *upd3* levels in the heart, KD of *lsn* led to a significant increase in *upd3* levels, whereas suppression of *ATPsynC* resulted in trending towards an increase in *upd3* expression (*P*=0.0719) (Fig. 6C). As another measure of inflammatory-like state upon cardiac KD, we performed hemocyte counts. Similarly, cardiac-specific KD of *lsn* led to a significantly increased number of hemocytes in the hemolymph (Fig. S11B). Furthermore, cardiac-specific KD of *lsn* also resulted in a significant increase in *upd3* levels in the head, while that of *ATPsynC* resulted in an increase that was not significant (Fig. 6D, *P*=0.33). Together, these data suggest that the inflammatory state of the heart, indicated by elevated *upd3* levels, mediates the non-cell-autonomous effects of *lsn* on sleep.

**Fig. 7.**
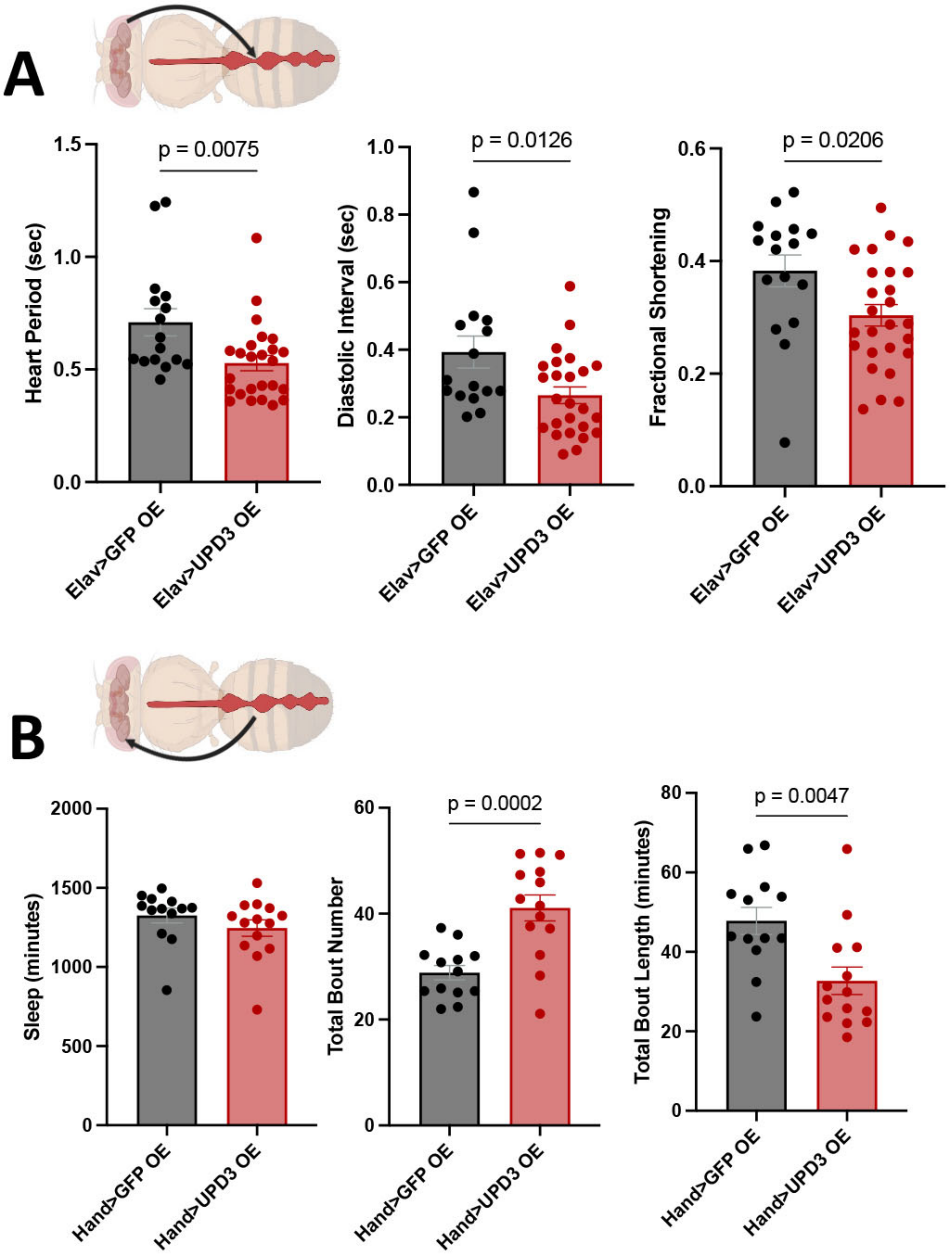
**Overexpressing Upd3 in neurons or the heart leads to cardiac or sleep dysfunction, respectively.** (A) Bar graphs showing cardiac physiological parameters – heart period (left), diastolic interval (middle) and fractional shortening (right) – in 3-week-old male flies with neuronal-specific overexpression (OE) of Upd3 (*n*=16-26 per group). (B) Bar graphs showing quantitative sleep parameters – total sleep amount (left), total bout number (middle) and total bout length (right) – in 3-week-old male flies with cardiac-specific overexpression of Upd3. *n*=13-14 per group. Each data point represents one fly. Statistics were calculated by unpaired two-tailed Student's *t*-test. Created in BioRender by Abou Daya, F. (2025). https://BioRender.com/6kvmu5y. This figure was sublicensed under CC-BY 4.0 terms.

To assess whether elevated Upd3 levels in either tissue are sufficient to non-cell-autonomously impact cardiac function or sleep, we overexpressed Upd3 in neurons using *elav-Gal4* or the heart using *Hand-Gal4*. Overexpression of Upd3 in the brain resulted in an increase in total sleep amount (Fig. S12A), while also resulting in a tachycardiac phenotype, similar to that resulting from neuronal *Imp* KD (Fig. 5B,D), characterized by decreased HP, DI and cardiac performance reflected by decreased FS (Fig. 7A). Overexpression of Upd3 in the heart resulted in cardiac dysfunction similar to that resulting from cardiac KD of *lsn*, characterized by increased AI, DD and SD, and decreased FS (Fig. S12B). Although cardiac overexpression of Upd3 did not affect overall sleep quantity, increased Upd3 in the heart did result in increased sleep fragmentation, with flies having significantly more sleep bouts that were significantly shorter in length (Fig. 7B), similar to those resulting from cardiac KD of *ATPsynC* and *lsn* (Fig. 5E-G). Together, these data demonstrate that increased Upd3 levels in neurons are sufficient to cause cardiac dysfunction, while elevated Upd3 in the heart is sufficient to cause increased sleep fragmentation. These findings indicate that inflammation, mediated by Upd3 in flies, is a bidirectional mechanism underlying the link between sleep and CVD.

## DISCUSSION

This study is the first to identify four genes at a single locus that link CVD and insomnia: *ATPsynC*, *Bruce*, *lsn* and *Imp* (*ATP5G1*, *UBE2Z*, *SNF8* and *IGF2BP1* in humans). Genetic screens have been previously applied in different model systems to identify genes involved in different CVDs or sleep regulation (Koh et al., 2008; Neely et al., 2010; Kamp et al., 2010; Snider and Conway, 2011; Spielmann et al., 2022; Lenz et al., 2015; Palermo et al., 2023). Despite the numerous advantages of these models for functional and behavioral screening, few studies have utilized them to test genes identified in human GWASs. A recent study (Palermo et al., 2023) used *Drosophila* to identify causal variants reported in an insomnia GWAS (Jansen et al., 2019), including our insomnia-related locus, and screen candidate genes to pinpoint those involved in sleep regulation. However, there are no studies to date that identify genes related to both diseases or investigate functional genetic mechanisms underlying a connection between CVD and sleep dysfunction.

Here, we used an innovative approach integrating the use of human genetics in conjunction with fly genetics to identify genes related to each disease and advance the understanding of the association between CVD and insomnia. We focused on a genetic locus identified in both CVD and insomnia GWASs (Jansen et al., 2019; Watanabe et al., 2020; van der Harst and Verweij, 2018), and identified *Drosophila* orthologs of potential nearby causal genes (Fig. 1). The locus we identified presented as a colocalization signal for both diseases. Interestingly, of 554 risk loci for insomnia identified thus far, this locus is among only three loci that colocalize with CVD (pp>0.90; others include the *APOE* region and *LINGO4/RORC*) (Watanabe et al., 2022). Functional dissection of other association signals for cardiovascular/cardiometabolic traits in this same genomic region suggests the importance of this region in CVD and the complex contributions of multiple genes at the locus to CVD pathogenesis (Erbilgin et al., 2013).

The first objective of our study was to functionally demonstrate a novel role of these genes in CVD and/or insomnia. Thus, we performed tissue-specific, neuronal or cardiac, KD of each gene. Although KD of *Imp* significantly increased sleep amount and decreased sleep quality, it also decreased the activity and speed of flies, thus suggesting an activity-dependent effect on sleep (Fig. 2). Moreover, upon KD of *Imp*, there was strong circadian disruption, which could, in turn, contribute to the lower activity levels and increased sleep as the circadian clock is a major regulator of sleep. Therefore, future studies testing different levels of *Imp* KD and their effects on sleep, activity and circadian rhythms are required for a better understanding of the specific role of *Imp* in sleep physiology. Even though neuronal KD of *Imp* significantly affected sleep, cardiac KD did not have a cell-autonomous effect on cardiac performance but only led to a tachycardic phenotype (Fig. 3).

Neuronal suppression of *ATPsynC* only increased overall sleep duration (Fig. 2), which is supported by published findings in another study screening insomnia-related genes identified from GWASs (Palermo et al., 2023). This increased sleep corresponded with decreased locomotor activity, which has been recently reported in humans with *ATP5G1* variants and flies with *ATPsynC* mutations (Neilson et al., 2022). Video tracking of flies to probe for locomotion speed showed that speed was decreased only in males even though both male and female flies showed an increase in sleep amount, suggesting that the effect of *ATPsynC* on sleep is independent of locomotion defects (Fig. 2; Fig. S4). Moreover, cardiac suppression of *ATPsynC* significantly compromised cardiac function characterized by severely increased arrhythmia, disrupted structure and fibrosis (Figs 3 and 4). Both cardiac and neuronal KD of *ATPsynC* also increased *upd3*-specific inflammation (Figs 4 and 6), which is an important indicator of cardiac injury and has been associated with sleep dysfunction (Dzierzewski et al., 2020). These findings revealed a novel role of *ATPsynC* in cardiac and sleep regulation in a cell-autonomous manner. Both the neurons and heart require large amounts of ATP to perform their functions. In both organs, ATP is essential for electrophysiological activities in resting and active states (Mandawat et al., 2021; Harding et al., 2023), and reduction of ATP levels impairs neural and cardiac functions (Mandawat et al., 2021; Henne et al., 2011; Oshima et al., 2016). ATP is produced by ATP synthase, and impairing the function of ATP synthase is known to alter ATP levels and hence lead to different cardiovascular and neurological diseases (Rock and Kono, 2008; Gera et al., 2022). *ATPsynC* is an important component of ATP synthase. Therefore, KD of *ATPsynC* may disrupt the function of ATP synthase, thus contributing to the CVD and sleep disruptions we observed.

Cardiac-specific KD of *lsn* also resulted in a dilated cardiomyopathy-like phenotype characterized by significant dilation, compromised cardiac performance and evident myofibril disorganization (Figs 3 and 4). Interestingly, patients with dilated cardiomyopathy also develop inflammation and cardiac fibrosis (Mandawat et al., 2021; Harding et al., 2023), which was also observed in *lsn* flies (Fig. 4). *lsn* hearts also showed a unique non-beating, heart failure-like phenotype that worsened with age (Fig. 3). These novel findings establish a cell-autonomous role for *lsn* in cardiac dysfunction. *lsn* is part of the endosomal sorting complex required for transport (ESCRT) pathway, which is a key mechanism of multivesicular body (MVB) biogenesis (Xu et al., 2017; Xie et al., 2019). MVBs form exosomes, which are crucial for intercellular communication and have been implicated in the pathophysiology of CVD and other diseases (Xu et al., 2017; Xie et al., 2019; Marebwa et al., 2018). Disruption of the ESCRT pathway impairs MVB function, leading to the accumulation of damaged proteins that should be degraded. This buildup causes cellular stress and can trigger cell death (Henne et al., 2011; Oshima et al., 2016). This may, in turn, induce an inflammatory response, potentially through Upd3, which then can upregulate the production of Pericardin and, eventually, fibrosis (Rock and Kono, 2008; Gera et al., 2022). This supports the importance of *lsn* in cardiac function we observed.

Our next objectives were to assess associations between CVD and insomnia, and to assess the effects of one disease on the other through *ATPsynC*, *Bruce*, *lsn* and *Imp* (Fig. 5). First, we suppressed these genes neuronally and assessed cardiac function. Unlike cardiac KD, neuronal suppression of *Imp* alone significantly reduced cardiac function in a non-cell-autonomous manner, while also producing strong cell-autonomous sleep phenotypes. One possible mechanism reported to underlie the effects of sleep dysfunction on cardiac function is inflammation (Javaheri and Redline, 2017). Notably, neuronal KD of *Imp* increased inflammation in the heart (Fig. 6). Furthermore, the overexpression of Upd3 in neurons phenocopied those non-cell-autonomous effects on cardiac function (Fig. 7), further suggesting the influence of sleep dysfunction on cardiovascular performance through inflammation, supporting Mendelian randomization reports that show an effect of insomnia on CVD (Lane et al., 2019; Watanabe et al., 2022).

Although previous observational and genetic studies more commonly report an effect of sleep on CVD, some human studies show an effect of heart failure on sleep interruption (Zheng, 2021). Therefore, we were interested in assessing whether there is an influence in the opposing direction, from the heart on the neurons. Therefore, we suppressed *ATPsynC*, *Bruce*, *lsn* and *Imp* in the heart and assessed sleep physiology (Fig. 5). We observed non-cell-autonomous effects on sleep in genes with cardiac dysfunction upon cardiac-specific KD, where flies with cardiac KD of *lsn* and *ATPsynC* had increased sleep fragmentation. Moreover, although cardiac KD of *Bruce* did not affect heart function in 1-week-old flies, it significantly reduced cardiac function in 3-week-old flies (Fig. S9), which in turn increased sleep amount non-cell-autonomously. This shows an effect of cardiac dysfunction on sleep. Overall, these findings suggest a non-cell-autonomous influence of *ATPsynC*, *lsn* and *Bruce* on sleep regulation.

Although the influence of heart function on the nervous system has been reported, the underlying mechanisms remain poorly understood (Xie et al., 2019; Marebwa et al., 2018). We hypothesized again that inflammation is a mechanism underlying the effects of cardiac dysfunction on sleep disruption. KD of *lsn* and *ATPsynC* increased inflammation in the head upon cardiac suppression (Fig. 6). Importantly, we also found that the overexpression of *upd3* in the heart leads to cardiac dysfunction cell-autonomously and, in turn, sleep disruption non-cell-autonomously (Fig. 7; Fig. S12), recapitulating the non-cell-autonomous effects of cardiac KD of *ATPsynC* or *lsn* on sleep observed. Overall, our results suggest inflammation through *upd3* as an important underlying factor in the bidirectional connection between cardiovascular function and sleep physiology.

One limitation of our study is the inability to characterize vascular and atherosclerotic phenotypes in the fly as the rs4643373 locus was linked to CAD in previous GWASs. However, to address this, we measured inflammation, which has a crucial role in CAD (Christodoulidis et al., 2014), to further characterize the cardiac phenotypes observed upon KD of genes near the CVD- and insomnia-related locus. Our study provides an important basis for future studies in more complex model systems to further characterize the roles of these genes in cardiovascular function. Moreover, our study focused on the effects of suppression of these genes on sleep and cardiac physiology. Future work will evaluate the effects of overexpressing these genes on each tissue and assess the subsequent cell-autonomous and non-cell-autonomous effects on sleep and cardiac function.

**Fig. 8.**
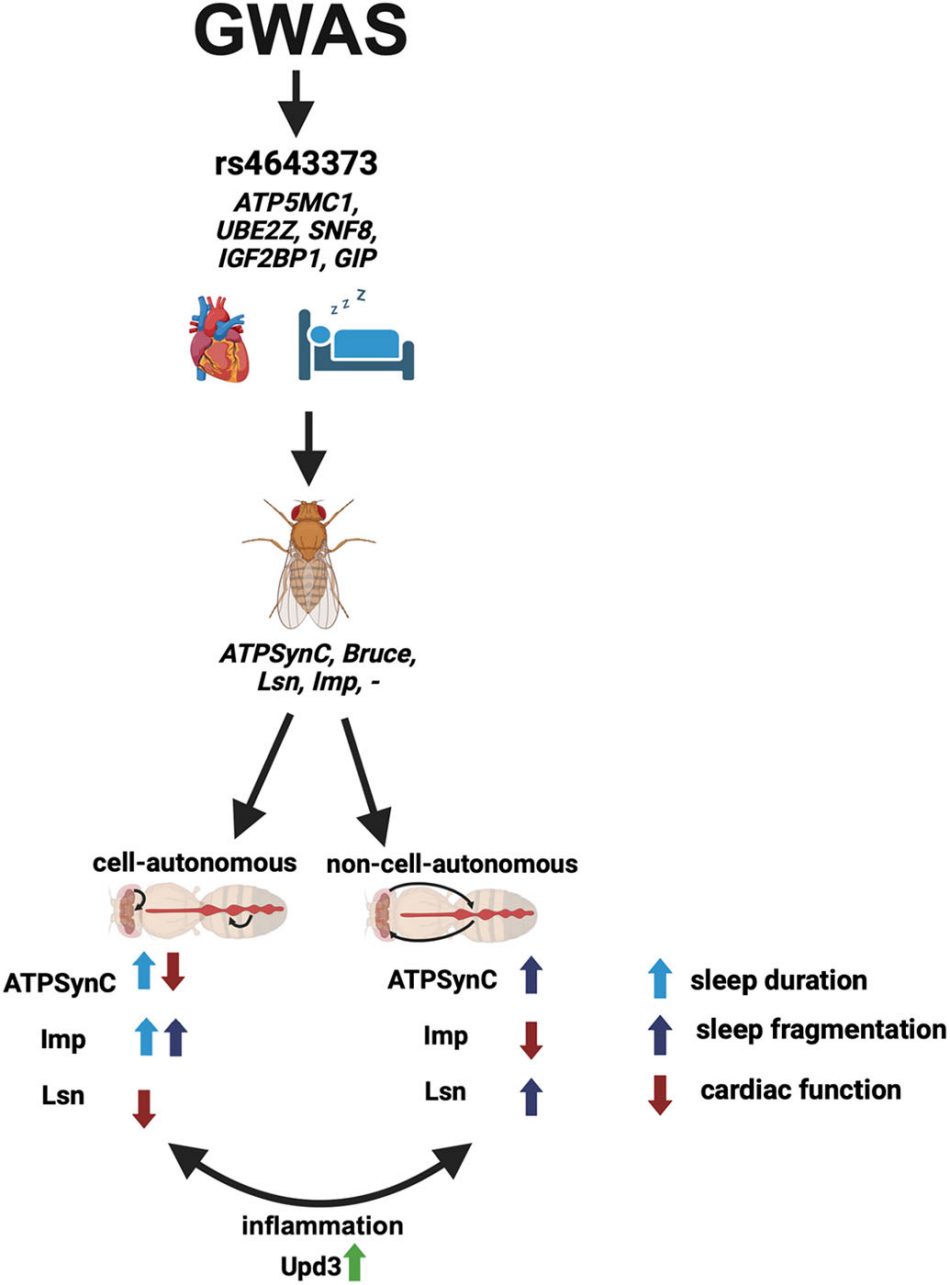
**Graphical summary displaying the main findings of this study.** Independent GWASs identified one genetic locus associated with CVD and insomnia. Near this locus, we identified five candidate genes, four of which have fly orthologs. To investigate the roles of genes near this locus in neurons and the heart, we suppressed their expression in each tissue and assessed their effects on sleep and cardiac function in a cell-autonomous or non-cell-autonomous manner. Our main findings include the following: one gene (*ATPsynC*) is involved cell-autonomously in sleep and cardiac function and affects sleep non-cell-autonomously through the heart; one gene is (*Imp*) involved cell-autonomously in sleep and affects cardiac function non-cell-autonomously through sleep; and one gene (*lsn*) is involved cell-autonomously in cardiac function and affects sleep non-cell-autonomously through the heart. We also found that cell-autonomous and non-cell-autonomous effects were accompanied by inflammation through elevation of *upd3* levels. Created in BioRender by Abou Daya, F. (2025). https://BioRender.com/s6m47bm. This figure was sublicensed under CC-BY 4.0 terms.

In summary, we report four important findings: first, *ATPsynC* is the only gene that is important cell-autonomously for both sleep and cardiac function and affects sleep non-cell-autonomously through the heart; second, *lsn* is important for cardiac function cell-autonomously and only affects sleep non-cell-autonomously through the heart; third, *Imp* is important cell-autonomously for sleep and affects the heart only non-cell-autonomously through sleep; and fourth, we functionally identified inflammation as a mechanism connecting cardiovascular and sleep dysfunction, bidirectionally (Fig. 8). These findings advance our understanding of the association between CVD and sleep disorders and provide a basis for future studies to help develop therapeutic strategies that prevent or attenuate insomnia and coincident CVD.

## MATERIALS AND METHODS

### LocusZoom plots and phenotypic associations for the rs4643373 region and eQTL analyses

The LocusZoom plots for the CVD and insomnia SNP, rs4643373, were generated using LocusZoom v1.3 (20 June 2014). Nearby associations with rs4643373 were obtained from the Cardiovascular Disease Knowledge Portal [accessed on 27 September 2023; https://cvd.hugeamp.org/variant.html?variant=rs4643373 (RRID:SCR_016536)]. Results were plotted using LDassoc in LDLink. The Genotype-Tissue Expression (GTEx) V8 database was used for multi-tissue eQTL analyses. The GTEx V8 data contain a total of 17,382 RNA-sequencing samples from 948 post-mortem donors. The GTEx data used for multi-tissue eQTL analyses described in this paper were obtained from the GTEx Portal on 8 March 2023.

### *Drosophila* stocks

*Drosophila* stocks were cultured at 25°C on standard agar media (Livelo et al., 2023). UAS-RNAi transgenic stocks of CVD- and insomnia-related genes were obtained from Vienna *Drosophila* Resource Center (VDRC) and Bloomington *Drosophila* Stock Center (BDSC): *ATPsynC*-RNAi [VDRC: 106834 (KK108875); BDSC: **35464** (GL00390), 57705 (HMC04894)], *lsn*-RNAi [VDRC: 110350 (KK100908), **21658** (GD10787); BDSC: 38289 (HMS01747)], *Bruce*-RNAi [VDRC: 107620 (KK100132), 48309 (GD16949); BDSC: **51814** (HMC03385)], *Imp*-RNAi [VDRC: 20321 (GD9232), 20322 (GD9232); BDSC: 38219 (GL00660), **55645** (HMC03794), 34977 (HMS01168)] and control lines [*w^1118^* (VDRC: 60100; BDSC: 36303), *Act5C-Gal4* (BDSC: 4414); *24b-Gal4* (BDSC: 1767) and *elav-Gal4* (BDSC: X, 458; II, 8765; GS, 43642)]. Throughout the paper, (+) refers to *w^1118^*. *Hand-Gal4* was obtained from Dr Olson's laboratory (University of Texas Southwestern Medical Center, Dallas, TX, USA). *UAS-upd3* was obtained from Dr Harrison's laboratory (University of Kentucky, Lexington, KY, USA). Genotypes are listed in Table S5. Data from RNAi lines were not combined if more than one RNAi line was used. Line 1 is underlined and is the main line used in all experiments. Line 2 is bolded and used only in Fig. S2. *ATPsynC* Line 2 was used for neuronal-specific suppression as Line 1 was lethal when crossed with *elav-Gal4*. *elav-Gal4* (II) was used in overexpression experiments to allow for observing more subtle phenotypes induced by lower expression of constructs, as overexpressing with *elav-Gal4* (X) was lethal.

### Ubiquitous and tissue-specific KD and genetic modulation

The *GAL4-UAS* system (Duffy, 2002) was used to drive the KD of CVD- and insomnia-related genes ubiquitously or tissue specifically. Adult flies possessing UAS RNAi CVD- and insomnia-related genes were crossed to *Hand-Gal4*, *elav-Gal4*, *Act5C-Gal4*, *Ubi-Gal4* or *24b-Gal4* flies and incubated at 25°C throughout development. Adult male and female F1 progeny were separated according to sex and allowed to age, with a new food source supplied every 3 days prior to assays of cardiac function. Age-matched adults from *w^1118^* (wild type), V60100 or BL36303 (VDRC and BDSC RNAi controls) were crossed with each of the *Gal4* drivers as controls. Male and female flies were screened at 1 and 3 weeks of age for cell-autonomous assays and 3 weeks for non-cell-autonomous assays in at least two independent experiments. All flies were kept at 25°C, 50% humidity in a 12 h light (L):12 h dark (D) cycle.

### Sleep–wake behavioral and rhythmicity analysis

Three- to 4-day-old male and female progeny of *elav-Gal4* (cell-autonomous) and 2.5-week-old male progeny of *Hand-Gal4* (non-cell-autonomous) with control and RNAi lines of each of the four genes were collected, and individual flies were loaded into glass tubes containing standard fly food (*n*>16). Sleep–wake behavior was recorded using the DAM (TriKinetics) system in a 12 h L:12 h D cycle at 25°C. *Drosophila* activity (or wake) is measured by infra-red beam crosses in the DAM system (Feng et al., 2018). To evaluate the role of CVD- and insomnia-related genes in maintaining adult rhythms, 3- to 4-day-old male flies were loaded into glass tubes and entrained for at least 3 days to a 12 h L:12 h D cycle, followed by 3 days in constant darkness. Rhythmicity analysis was performed for each 3-day period in constant darkness. Data were analyzed using ClockLab and RStudio. Custom R scripts and methodology used with RStudio can be found at https://github.com/jameswalkerlab/Gill_et.al.. One-way ANOVA with Dunnett's multiple comparisons test for DAM system data was performed using GraphPad Prism. *Drosophila* sleep was defined by a period of at least 5 min of inactivity, demonstrated by zero beam breaks recorded (Hendricks et al., 2000). Average sleep per 24 h (ZT0-ZT24) of each genotype was calculated. Five days were used for analysis of 1-week-old flies, and 3 days were used for 3-week-old fly experiments to overcome decreased viability in older flies. Sleep bouts were quantified by counting the number of periods of sleep as defined above. Sleep fragmentation was defined by either the number of 1-min wakes or sleep bouts during a 24-h period. Data for daytime sleep are from ZT0 to ZT12 and that for nighttime sleep are from ZT12-ZT24.

### MARGO locomotion monitoring

*Drosophila* locomotion was video monitored using the MARGO system for automated tracking (Werkhoven et al., 2019; Elya et al., 2023). Behavior boards were prepared by the addition of standard fly food at one end. Individual 3- to 5-day-old male and mated female flies were loaded into the behavior boards such that there was one fly per channel. Flies were video recorded for 3 days at 20°C. Collected data from days 2 and 3 were analyzed using a custom script in MatLab. Speed data were calculated by the change in location of the centroid of the tracked fly between frames. These data were collected at a frame rate of 4 Hz and an experimental setup such that 3.4667 pixels equal 1 mm. Using these values, pixels per frame were converted to mm s^−1^ values. Frames in which flies were immobile were excluded from speed calculations. Therefore, average speed measurements are representative of the average speed while flies are in motion. Average speed values are an average of speeds from days 2 and 3 and normalized to controls set up within the same behavior board.

### Cardiac physiological analyses of semi-intact *Drosophila* hearts

One-week-old male and female progeny of *Hand-Gal4* (cell-autonomous) and 2.5-week-old male progeny of *elav-Gal4* (non-cell-autonomous) with control and RNAi lines of each of the four genes were collected, and semi-intact hearts were prepared as described (*n*>30) (Fink et al., 2009; Melkani et al., 2013). Direct immersion optics were used in conjunction with a digital high-speed camera (at 200 frames s^−1^; Hamamatsu Flash 4 camera) to record 30-s movies of beating hearts; images were captured using HC Image (Hamamatsu). Cardiac function was analyzed from the high-speed movies using semi-automatic optical heartbeat analysis (SOHA) software that quantifies heart rate, heart period, diastolic and systolic diameters, diastolic and systolic intervals, cardiac rhythmicity and FS, and produces the Mechanical-mode records (Fink et al., 2009; Melkani et al., 2013).

### Cytological studies of adult hearts

Dissected hearts from 1-week-old adults were relaxed by a 1-min treatment with 5 mM EGTA in hemolymph and then fixed with 4% paraformaldehyde in PBS for 30 min as previously described (Melkani et al., 2013). Fixed hearts were stained with anti-Pericardin antibody overnight (5 μg/ml, 1:10; Developmental Biology Hybridoma Bank, University of Iowa) followed by Alexa488-phalloidin for 30 min (1:1000; U0281, Abnova), which stains F-actin containing myofibrils. Samples were then mounted with Diamond Antifade Mountant with DAPI (P36966, Thermo Fisher Scientific). Confocal images were taken with a Nikon A1R HD microscope (University of Alabama at Birmingham) at 10× for Pericardin quantification and 20× for representative images for phalloidin staining. Quantification of Pericardin area in the confocal images from three to five independent male hearts per genotype was performed by thresholding images in ImageJ, then percentage area was measured (Voskobiynyk et al., 2020).

### Adult-specific KD of genes

To induce adult-specific KD, we crossed flies from each genotype with either *Hand-GeneSwitch-Gal4* or *elav-GeneSwitch-Gal4* and put them on RU486-containing (mifepristone; 459980010, Thermo Fisher Scientific) food 3-4 days after eclosion. For crosses with *Hand-GeneSwitch-Gal4*, we used food containing 200 µM RU486; for crosses with *elav-GeneSwitch-Gal4*, we used food containing 500 µM RU486 to allow activation of the GeneSwitch system. We changed the food twice a week for 3 weeks, then we measured the cardiac and sleep physiology of 3-week-old male flies.

### Viability

Adult flies (*n*>100, males and females) with suppression of CVD- and insomnia-related genes and controls were collected on the day of eclosion from the pupal case, designated as day 0. Approximately 30 flies were placed in each vial and transferred to a new vial every 3-4 days. The numbers of surviving adults were counted twice a week. The numbers of surviving adults were compared to the original number of adults collected on day 0, and the percentage for each day was graphed (Melkani et al., 2013).

### Hemocyte counts

To evaluate inflammation, fly hemolymph was collected from *n*>100 (per replicate, three biological replicates) 1-week-old adult male flies with cardiac-specific suppression using *Hand-Gal4* by making an incision in the thorax of flies and centrifuging them (Hiroyasu et al., 2018). Hemocytes were then counted by staining the hemolymph with 1:1 Trypan Blue dilution and using a hemocytometer (Hiroyasu et al., 2018).

### Real-time quantitative PCR

Dissected male hearts (*n*=10-12 per biological replicate, three biological replicates) and heads (*n*=10, per biological replicate, three biological replicates) from 1-week-old flies were placed in RNA lysis buffer (Zymo Research) and flash frozen. RNA from heads was extracted using a Zymo Research Quick-RNA Microprep Kit with on-column DNase I digestion. RNA from hearts was extracted using an RNeasy kit (Qiagen). Quantitative PCR was performed using SsoAdvanced Universal SYBR Green Supermix (Bio-Rad) in a Bio-Rad CFX Opus Real-Time PCR System. Expression was normalized with 60S ribosomal protein (*RpL11*). Primers for quantitative PCR are as as follows: *ATPsynC*-F, 5′-GCAACAGTCGGTGTCGCT-3′; *ATPsynC*-R, 5′-AGGCGAACAGCAGCAGGAA-3′; *lsn*-F, 5′-TCACCAAGGAGGACATCCTAATGG-3′; *lsn*-R, 5′-TCCGGGAATGGACTGAACTATGTA-3′; *Bruce*-F, 5′-AATAGCGCTCCATCTCGACCAT-3′; *Bruce*-R, 5′-ATCGACCATGCACAATGCTGT-3′; *Imp*-F, 5′-AATTCGCCGACCTGGAACTCT-3′; *Imp*-R, 5′-ACTCGACACCGTTCAGACCAA-3′; *upd3*-F, 5′-AGCCGGAGCGGTAACAAAA-3′; *upd3*-R, 5′-CGAGTAAGATCAGTGACCAGTTC-3′; *RpL11*-F, 5′-CGATCTGGGCATCAAGTACGA-3′; *Rpl11*-R, 5′-TTGCGCTTCCTGTGGTTCAC-3′. Results are presented as 2^−ΔΔCt^ values normalized to the expression of *RpL11* and control samples. All reactions were performed using biological triplicates. The means and s.e.m. were calculated in GraphPad Prism 9 software.

### Statistical analysis

For all quantitation except transcript levels and lifespan analyses, statistical significance was determined using one-way analysis of variance (ANOVA) followed by Dunnett's post-hoc test to determine significance between groups for sleep and cardiac physiological parameters. Comparisons to UAS controls were calculated by one-way ANOVA followed by Šidák's post-hoc test to assess the off-target effects of RNAi lines. Additional comparisons of sleep and cardiac parameters of flies to their respective RNAi controls in Table S6 were calculated by one-way ANOVA followed by Šidák's post-hoc test. For expression of transcript levels in heads, statistics were calculated by one-way ANOVA. For expression of transcript levels in hearts, statistics were calculated by Kruskal–Wallis test and without correcting for multiple comparisons to account for variability. For overexpression experiments, statistical significance was determined using an unpaired two-tailed Student's *t*-test. Bar graphs show mean±s.e.m. For comparisons between percentages of beating hearts at 1 versus 3 weeks of age, Fisher's exact test was performed. For lifespan studies, data were analyzed using the Kaplan–Meier test followed by multiple comparisons between control and experimental groups. Significance was presented using *P*-values in figures. All statistical analyses were performed with GraphPad Prism 9.

## Supplementary Material

10.1242/dmm.052139_sup1 Supplementary information
