# Supplementary material for: Identifying links between cardiovascular disease and insomnia by modeling genes from a pleiotropic locus
Source: Dis Model Mech. 2025 May 6;18(5):dmm052139. doi: 10.1242/dmm.052139 (PMC12140649; doi:10.1242/dmm.052139)
Supplement: Supplementary information [file dmm-18-052139-s1.pdf]

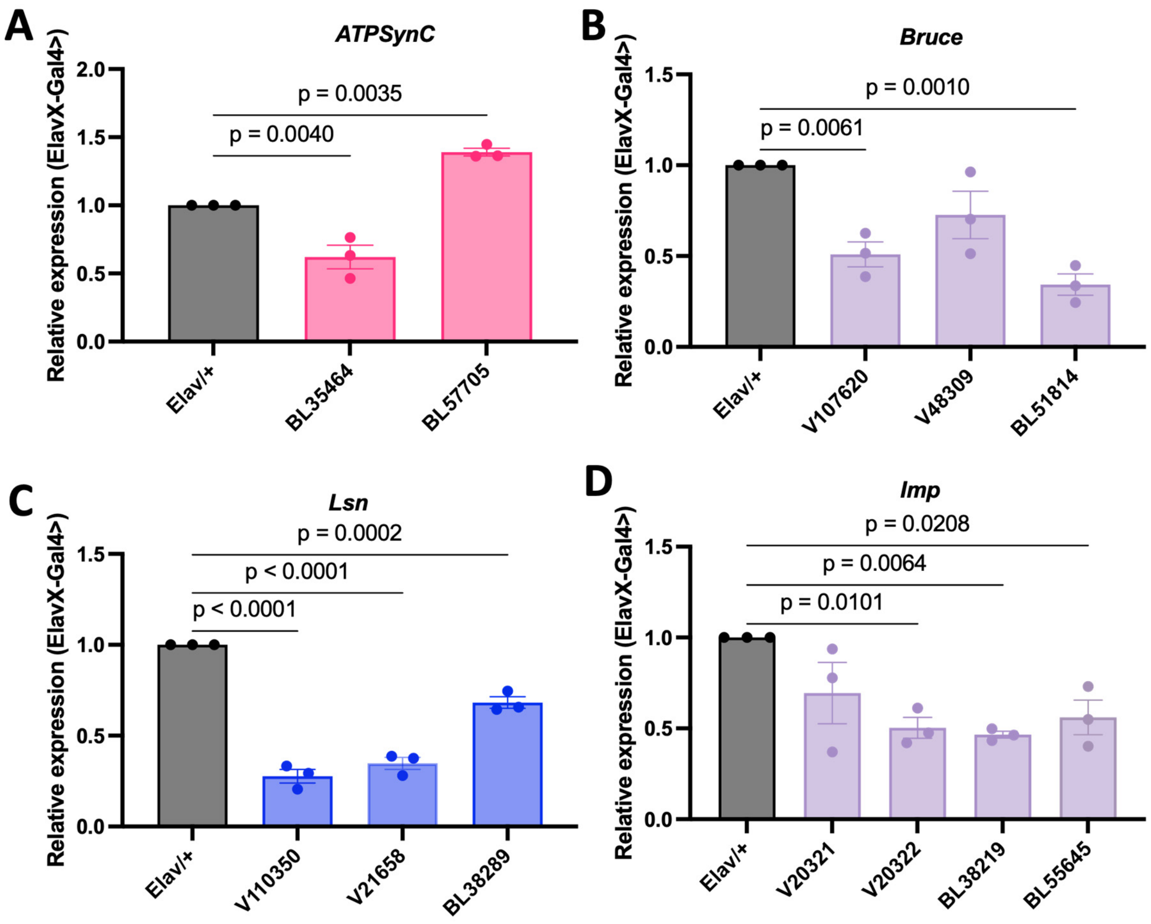

Fig. S1. Transcript levels of CVD and insomnia-related genes upon neuronal knockdown. Quantification of RNA levels of ATPSynC (A), Bruce (B), Lsn (C), and Imp (D) from heads of 1-week-old male flies with following neuronal-specific suppression. Each point represents 10-12 heads. Missing lines from A and D were lethal when crossed with *Elav-Gal4*. Statistics were calculated by 1-way ANOVA.

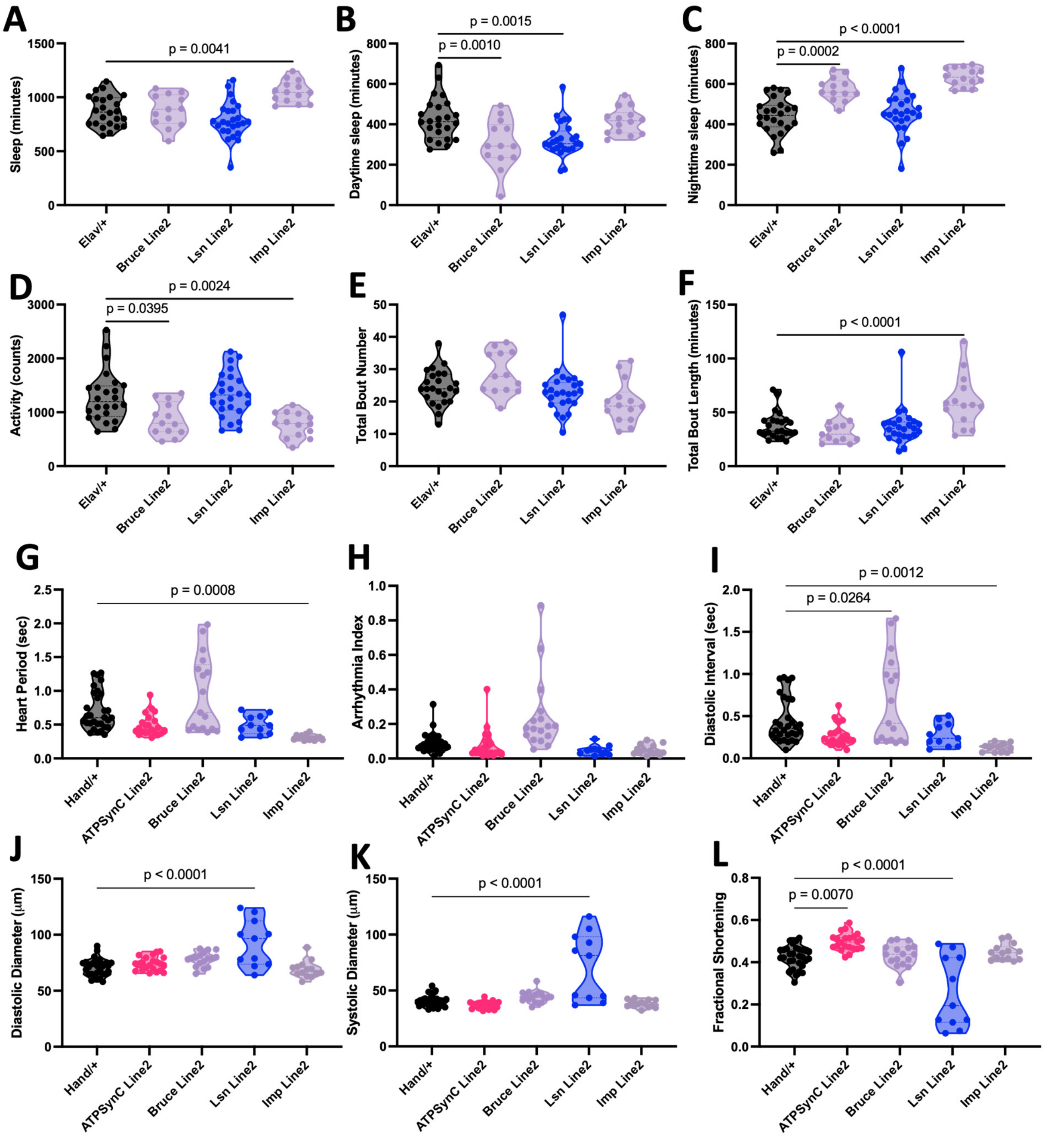

Fig. S2. Sleep and cardiac physiological parameters of secondary RNAi line for each gene. Violin plots for quantitative sleep parameters; sleep amount (A-C), locomotor activity (D), total bout number (E) and total bout length (F) from 1-week-old male *Drosophila* with neuronal-specific knockdown of CVD- and insomnia-related genes (N=12-41 per group). Line 2 for each gene is: *ATPSynC* (BL35464), *Bruce* (BL51814),

Lsn (V21658), and *Imp* (BL55645). *ATPSynC* Line 1 was lethal with *Elav-Gal4*. Violin plots for cardiac physiological parameters, heart period (G), arrythmia index (H), diastolic interval (I), diastolic diameter (J), systolic diameter (K), fractional shortening (L) from 1-week-old male flies with cardiac-specific knockdown of CVD- and insomnia-related genes (N=11-30 per group as shown in each panel, from at least 2 independent experiments). Each data point represents one fly. Statistics were calculated by 1-way ANOVA.

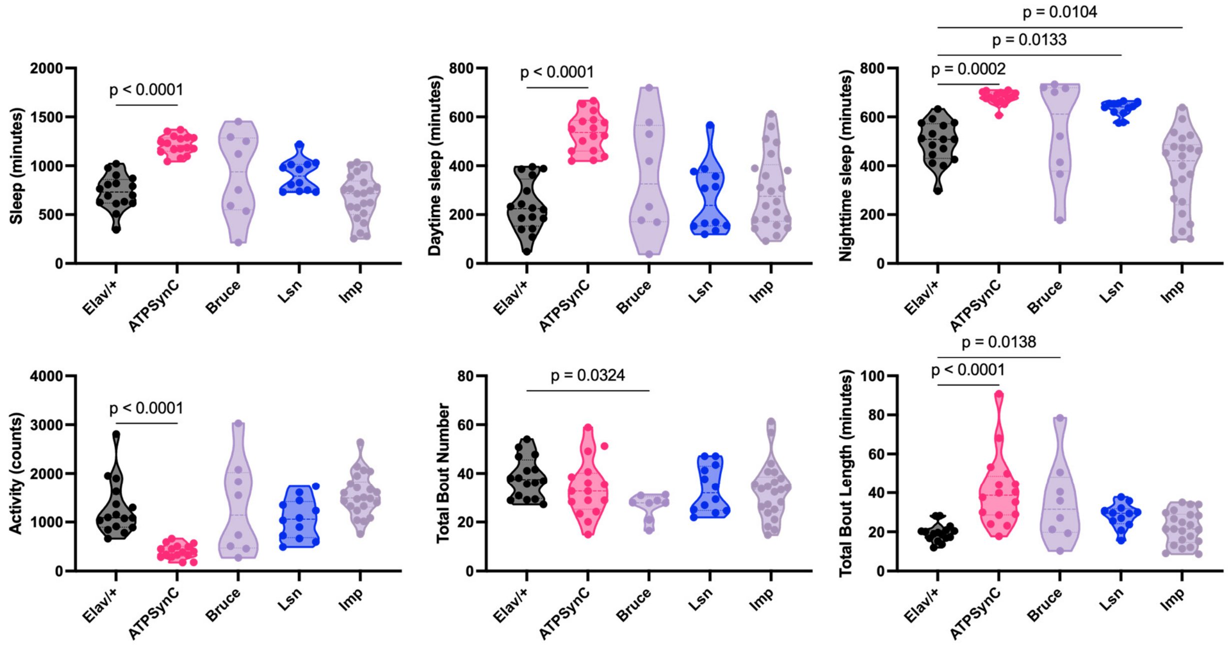

Fig. S3. Neuronal-specific suppression of CVD- and insomnia-related genes leads to sleep dysfunction in female flies. Violin plots for quantitative sleep parameters; total sleep amount, daytime sleep, nighttime sleep, total locomotor activity, total bout number and total bout length from 1-week-old female *Drosophila* with neuronal-specific knockdown of CVD- and insomnia-related genes (N=8-22 per group). Each data point represents one fly. Statistics were calculated by 1-way ANOVA.

Disease Models & Mechanisms • Supplementary information

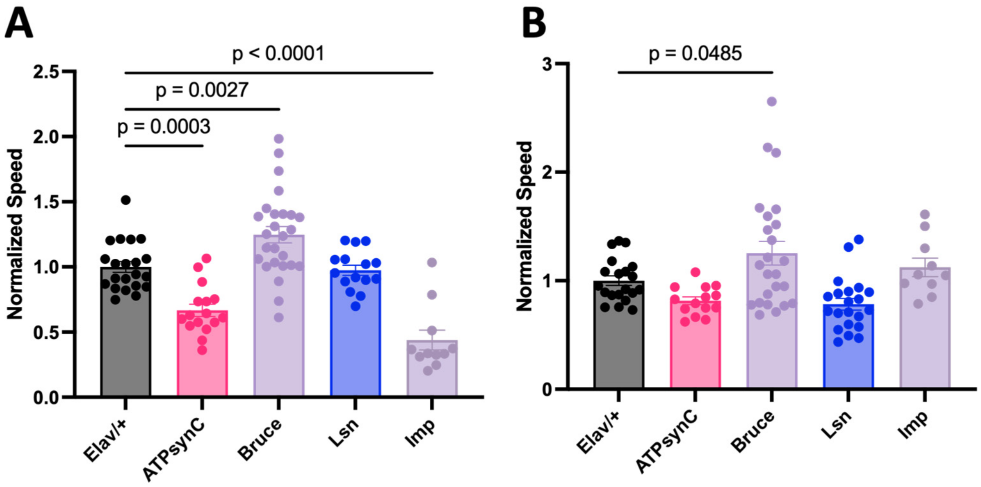

Fig. S4. Neuronal-specific suppression of CVD- and insomnia-related genes affects locomotion speed. Normalized locomotion speed of flies of male (A) and female (B) flies with neuronal RNAi knockdown of CVD- and insomnia-related genes with *Elav-Gal4* (n=11-26) as determined by MARGO. 1-week-old flies used. Each data point represents a fly. Statistics were calculated by one-way ANOVA for comparison to controls.

Disease Models & Mechanisms • Supplementary information

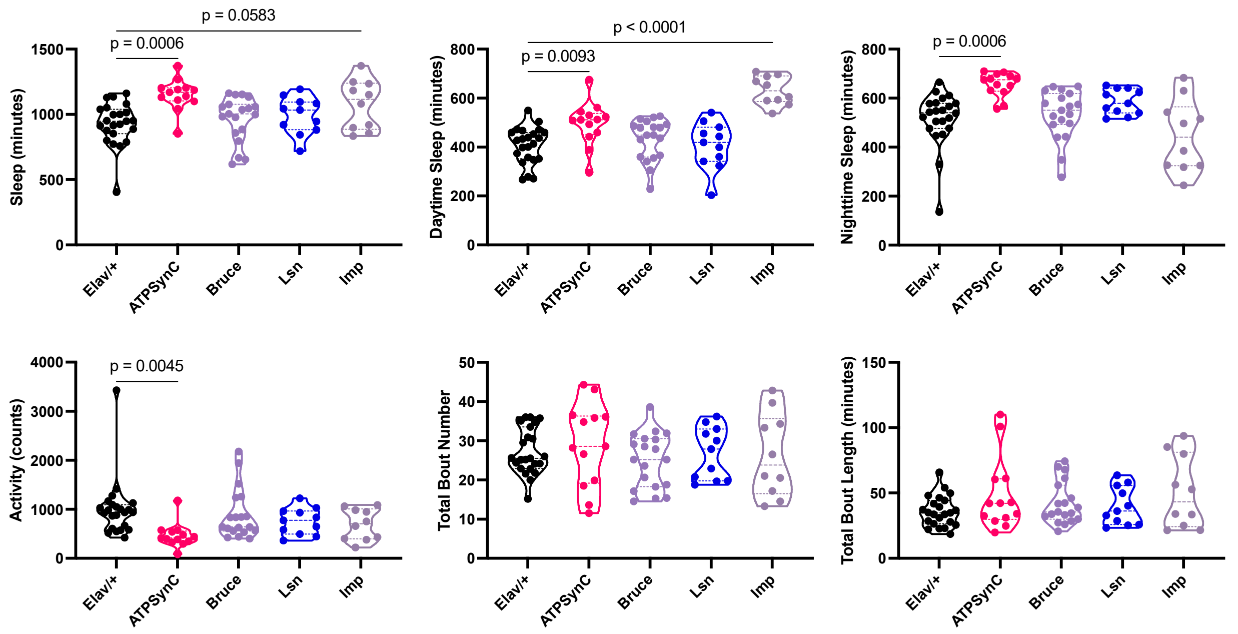

Fig. S5. Neuronal-specific suppression of CVD- and insomnia-related genes leads to sleep dysfunction in 3-week-old flies. Violin plots for quantitative sleep parameters; total sleep amount, daytime sleep, nighttime sleep, total locomotor activity, total bout number, and total bout length from 3-week-old male *Drosophila* with neuronal-specific knockdown of CVD- and insomnia-related genes. Each data point represents one fly. Statistics were calculated by 1-way ANOVA.

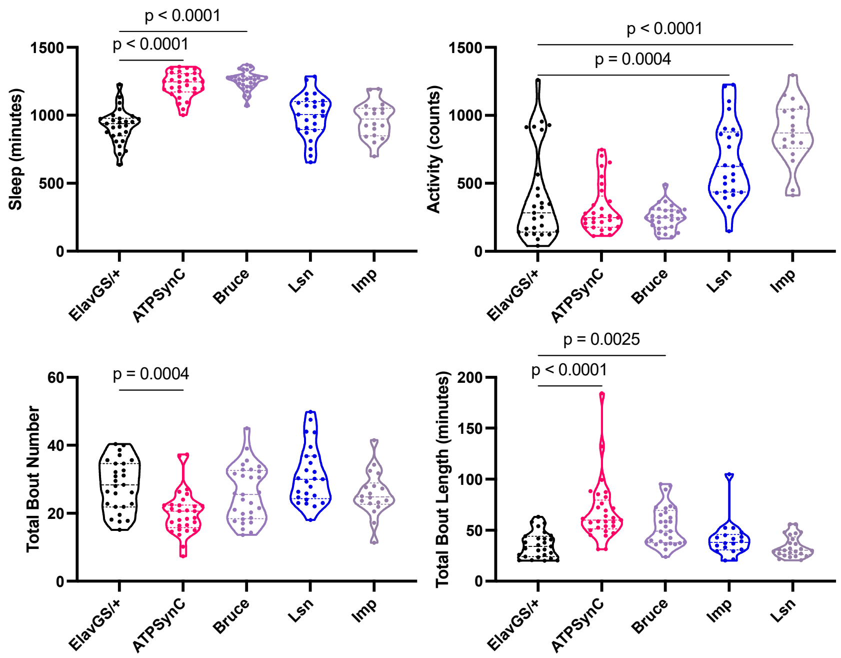

Fig. S6. Adult-specific neuronal-specific suppression of CVD- and insomnia-related genes compromise sleep. Violin plots for quantitative sleep parameters; sleep amount, locomotor activity, and total bout number and length from 3-week-old male *Drosophila* with adult-specific neuronal-specific knockdown of CVD- and insomnia-related genes using *Elav-GeneSwitch-Gal4*. Each data point represents one fly. Statistics were calculated by 1-way ANOVA.

Disease Models & Mechanisms • Supplementary information

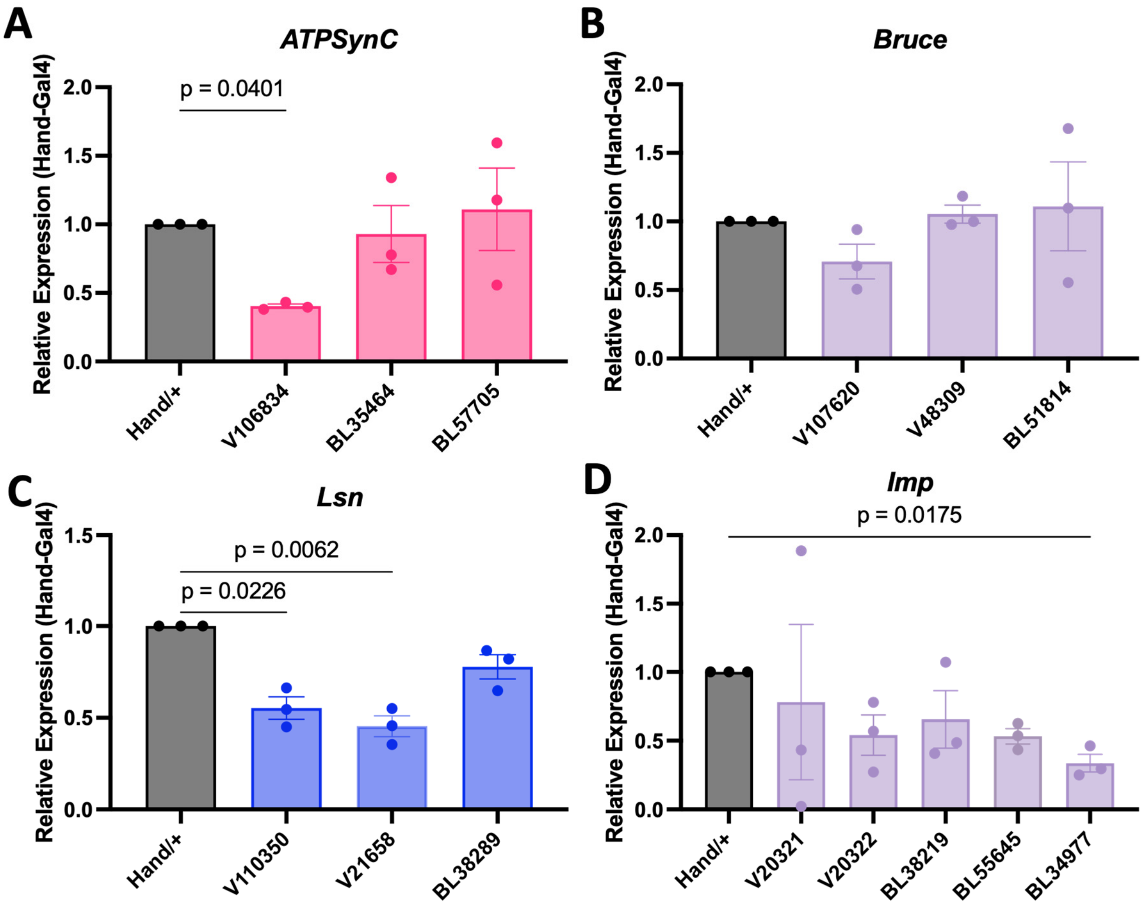

Fig. S7. Transcript levels of CVD and insomnia-related genes upon cardiac knockdown. Quantification of RNA levels of ATPSynC (A), Bruce (B), Lsn (C), and Imp (D) from hearts of 1-week-old male flies following cardiac-specific suppression. Each point represents 10-12 hearts. Statistics were calculated by the Kruskal-Wallis test and without correcting for multiple comparisons to account for variability.

Disease Models & Mechanisms • Supplementary information

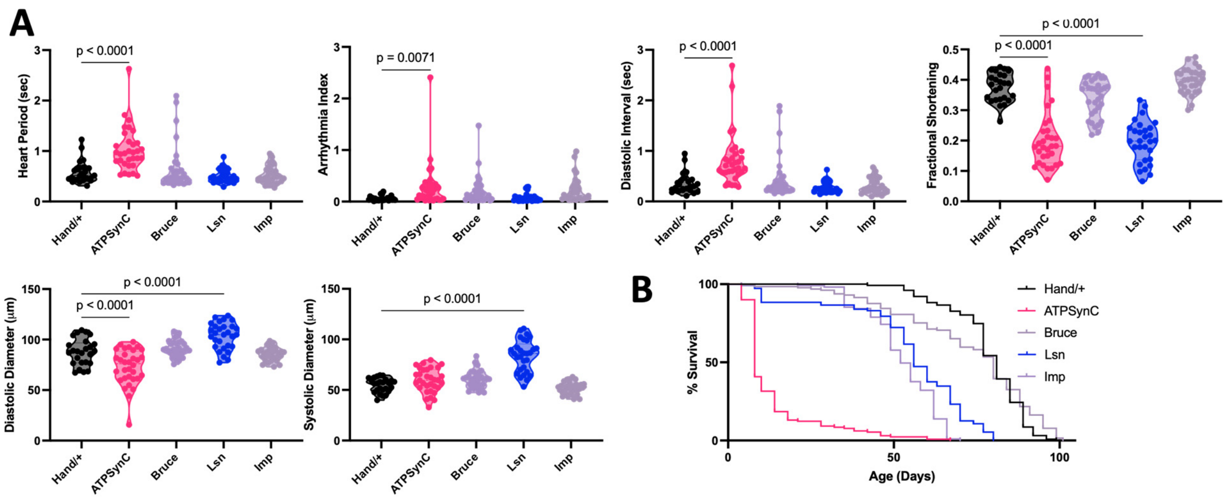

Fig. S8. Cardiac-specific suppression of CVD- and insomnia-related genes leads to cardiac dysfunction in female flies. Violin plots for cardiac physiological parameters, heart period, arrythmia index, diastolic interval, diastolic diameter, systolic diameter, and fractional shortening (A) from 1-week-old female flies with cardiac RNAi knockdown of CVD- and insomnia-related genes with Hand-Gal4 (N=25-32 per group). Each data point represents one fly. Lifespan assay (B) for female flies with cardiac RNAi knockdown of CVD- and insomnia-related genes with *Hand-Gal4* resulted in a significant decrease in lifespan (p<0.0001) of ATPSynC, Lsn, and Imp, but a nonsignificant change of Bruce. Graph plots % survival (n>100 for each group) vs. time post-eclosion. Statistics were calculated by 1-way ANOVA for A and a Kaplan-Meier test was performed for B.

Disease Models & Mechanisms • Supplementary information

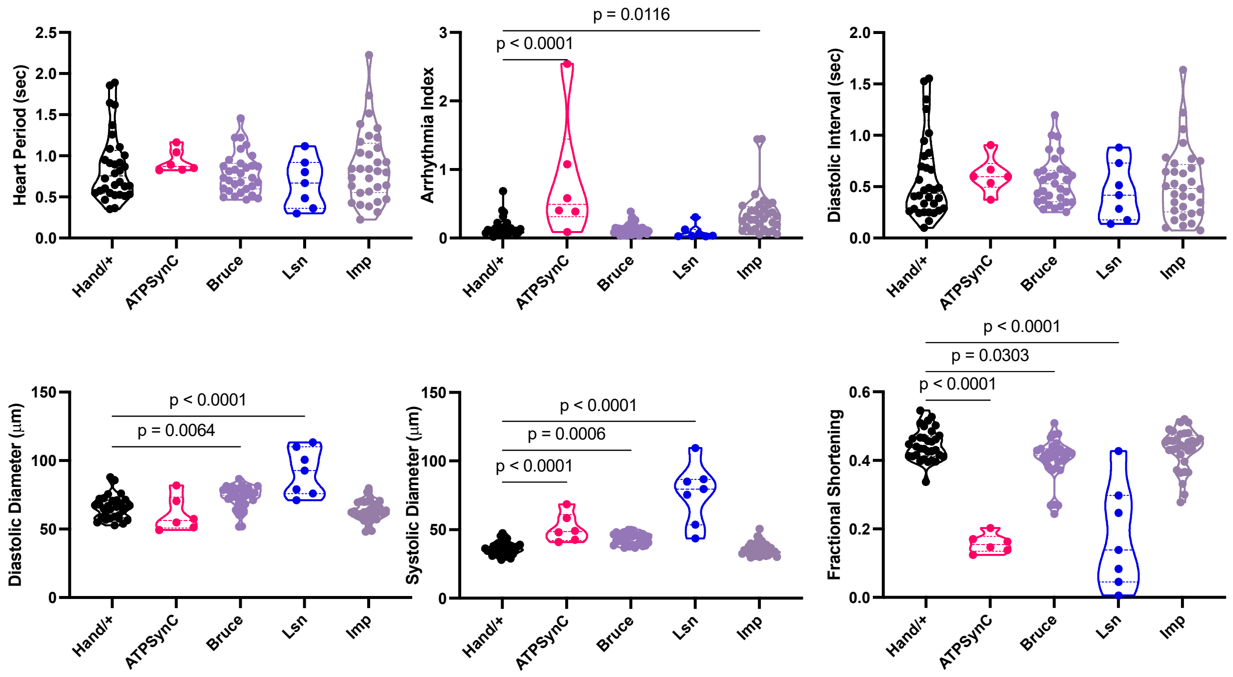

Fig. S9. Cardiac-specific knockdown of CVD- and insomnia-related genes leads to cardiac dysfunction in 3-week-old flies. Violin plots for cardiac physiological parameters, heart period, arrythmia index, diastolic interval, diastolic diameter, systolic diameter and fractional shortening from 3-week-old male flies with cardiac RNAi knockdown of CVD- and insomnia-related genes with *Hand-Gal4*. Each data point represents one fly. Statistics were calculated by 1-way ANOVA.

Disease Models & Mechanisms • Supplementary information

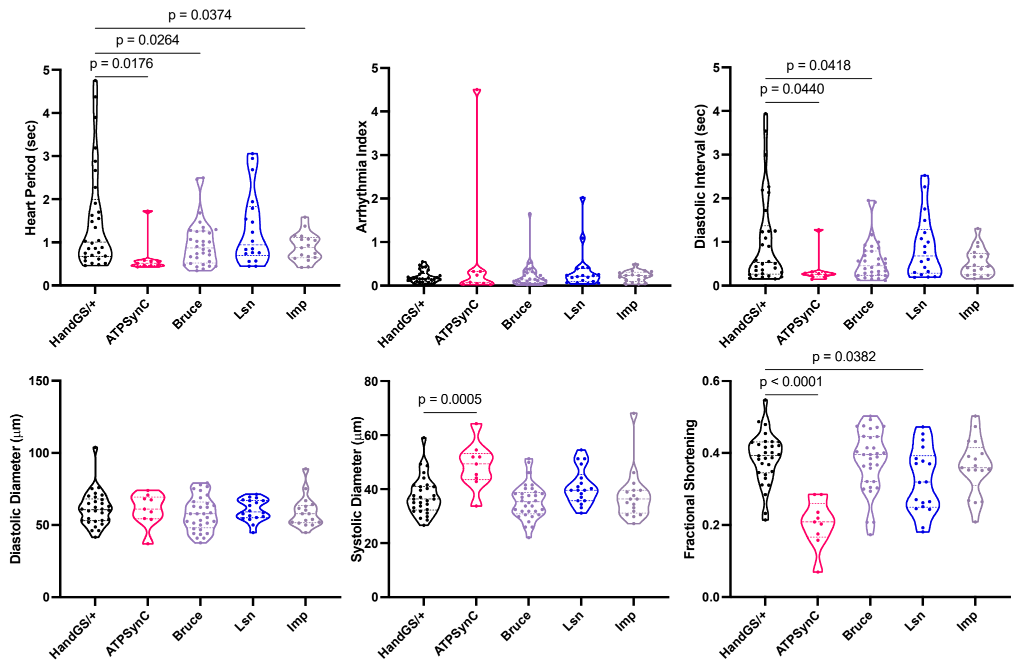

Fig. S10. Adult-specific cardiac-specific suppression of CVD- and insomnia-related genes compromise cardiac function. Violin plots for cardiac physiological parameters, heart period, arrythmia index, diastolic interval, diastolic diameter, systolic diameter and fractional shortening from 3-week-old male flies with adult-specific cardiac RNAi knockdown of CVD- and insomnia-related genes with *Hand-GeneSwitch-Gal4.* Each data point represents one fly. Statistics were calculated by 1-way ANOVA.

Disease Models & Mechanisms • Supplementary information

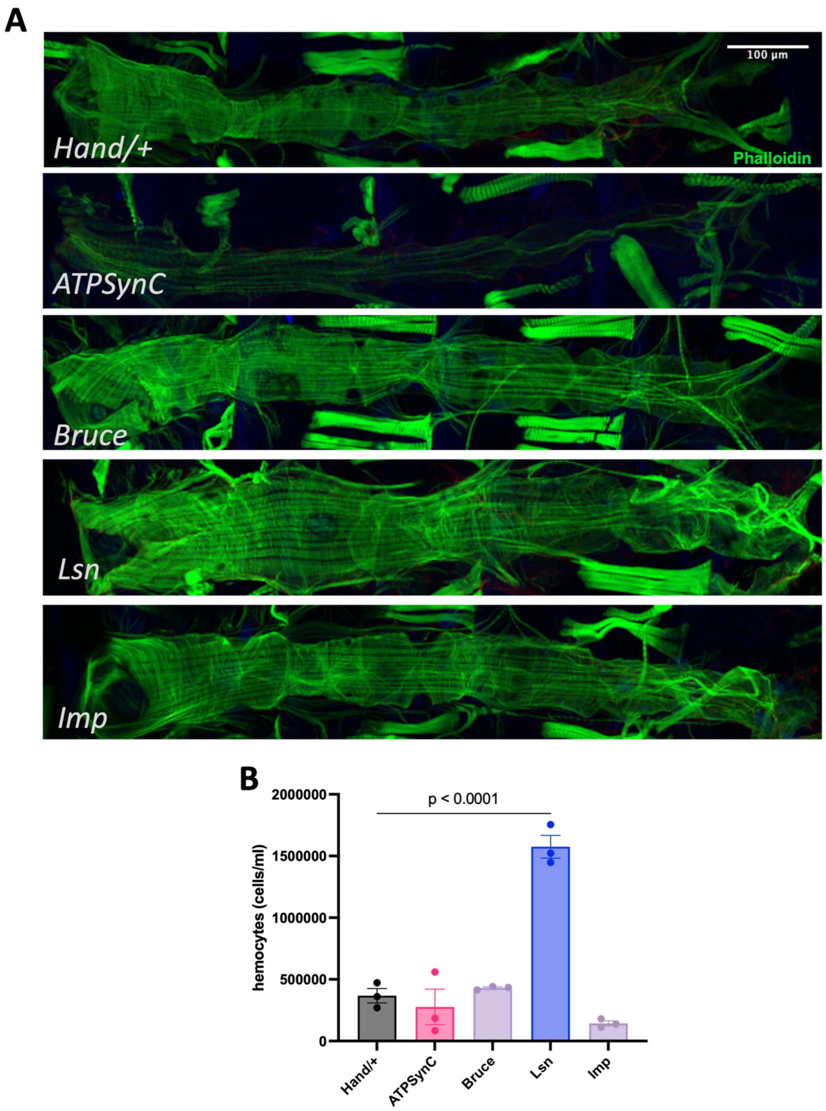

Fig. S11. Cardiac suppression of CVD- and insomnia-related genes influences cardiac cytology and inflammation. Representative images showing actin-containing myofibrils in whole hearts stained with Phalloidin from each group (A). Hemocyte counts (n=95-145 per data point per group) for each group (B). 1-week-old males were used. Statistics were calculated by 1-way ANOVA.

Disease Models & Mechanisms • Supplementary information

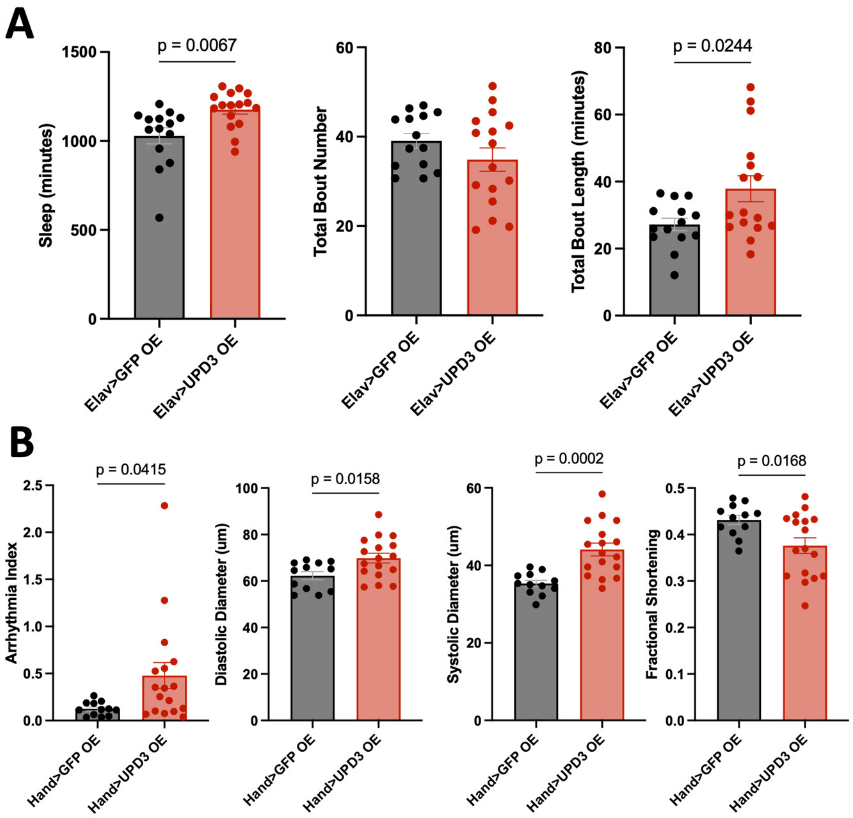

Fig. S12. Overexpressing Upd3 in the heart leads to cardiac dysfunction while overexpressing it in neurons increases sleep. Bar graphs showing quantitative sleep parameters; total sleep amount, total bout number, and total bout length from 3-week-old male flies with neuronal-specific overexpression of Upd3 (A). Bar graphs showing cardiac physiological parameters, arrythmia index, diastolic diameter, systolic diameter and fractional shortening from 3-week-old male flies with cardiac-specific overexpression of Upd3. N= 12-16 per group. Each data point represents one fly. Statistics were calculated by unpaired t-test.

Disease Models & Mechanisms • Supplementary information

Table S1. Multi-tissue eQTL analyses of CVD- and insomnia-related genes at rs4643373.

Gene	Tissue	Samples	NES	p-value	m-value
ATP5G1	Liver	208	0.0748	0.1	0.055
	Spleen	227	0.032	0.3	0
	Whole Blood	670	0.00858	0.6	0
	Skin - Sun Exposed (Lower leg)	605	0.00435	0.8	0.002
	Adipose Subcutaneous	581	0.00197	0.9	0.001
	Esophagus Mucosa	497	-0.0183	0.5	0.22
	Artery - Coronary	213	-0.019	0.6	0.502
	Lung	515	-0.0192	0.3	0.056
	Skin - Not Sun Exposed (Suprapubic)	517	-0.0239	0.3	0.144
	Adipose - Visceral (Omentum) Artery - Tibial	469	-0.0331	0.1	0.423
	Brain - Hippocampus	584	-0.0336	0.07	0.328
	Brain Caudate (basal ganglia)	165	-0.0339	0.3	0.656
	-	194	-0.0402	0.2	0.789
	Brain - Putamen (basal ganglia)	170	-0.0421	0.2	0.739
	Cells - EBV-transformed lymphocytes	147	-0.0443	0.4	0.751
	Kidney - Cortex	73	-0.0464	0.6	0.782
	Ovary	167	-0.0467	0.2	0.744
	Cells Cultured fibroblasts	483	-0.0482	1.90E-03	0.979
	Colon - Transverse	368	-0.0487	0.01	0.87
	Minor Salivary Gland	144	-0.0489	0.3	0.761
	Uterus	129	-0.0506	0.2	0.777
	Breast - Mammary Tissue	396	-0.0522	0.04	0.916
	Prostate	221	-0.0533	0.1	0.781
	Pancreas	305	-0.0614	0.04	0.95
	Brain - Anterior cingulate cortex (BA24)	147	-0.063	0.2	0.87
	Stomach	324	-0.069	0.008	0.985
	Esophagus Gastroesophageal Junction	330	-0.0698	0.01	0.963
	Muscle - Skeletal	706	-0.076	3.20E-08	1
	Artery - Aorta	387	-0.0772	0.007	1
	Adrenal Gland	233	-0.0825	0.02	0.947
	Small Intestine - Terminal Ileum	174	-0.0839	0.01	0.977
	Brain - Nucleus accumbens (basal ganglia)	202	-0.0887	0.005	0.994
	Vagina	141	-0.0897	0.02	0.963
	Thyroid	574	-0.0937	2.40E-06	1
	Brain - Cortex	205	-0.1	0.009	0.981
	Testis	322	-0.103	0.008	0.981
	Nerve - Tibial	532	-0.105	2.10E-07	1
	Colon - Sigmoid	318	-0.11	2.70E-04	1
	Heart - Atrial Appendage	372	-0.112	6.80E-06	1
	Esophagus - Muscularis	465	-0.121	2.50E-08	1
	Brain - Hypothalamus	170	-0.124	0.006	0.996
	Brain Frontal Cortex (BA9)	175	-0.127	1.00E-04	1
	Brain Cerebellar Hemisphere	175	-0.13	2.90E-03	1
	Brain - Amygdala	129	-0.139	0.007	0.987
	Pituitary	237	-0.154	2.10E-04	1
	Brain - Substantia nigra	114	-0.159	0.02	0.871
	Heart Left Ventricle	386	-0.164	2.20E-09	1
	Brain - Spinal cord (cervical c-1)	126	-0.172	2.10E-03	0.985
	Brain - Cerebellum	209	-0.223	8.00E-06	0.999

Gene	Tissue	Samples	NES	p-value	m-value
SNF8	Liver	208	0.0491	0.2	0.057
	Small Intestine - Terminal Ileum	174	0.0142	0.7	0.11
	Colon - Sigmoid	318	0.0017	1	0.068
	Kidney - Cortex	73	-0.0153	0.8	0.689
	Adipose - Visceral (Omentum)	469	-0.0158	0.4	0.006
	Skin - Sun Exposed (Lower leg)	605	-0.022	0.4	0.064
	Whole Blood	670	-0.0265	0.2	0.013
	Prostate	221	-0.0285	0.5	0.435
	Breast - Mammary Tissue	396	-0.0393	0.06	0.361
	Esophagus - Mucosa	497	-0.0426	0.2	0.676
	Colon - Transverse	368	-0.0481	0.1	0.705
	Muscle - Skeletal	706	-0.0567	0.01	0.963
	Minor Salivary Gland	144	-0.0627	0.3	0.889
	Lung	515	-0.0646	1.30E-03	0.989
	Artery - Tibial	584	-0.0729	8.70E-04	1
	Heart - Atrial Appendage	372	-0.0732	0.02	0.98
	Spleen	227	-0.0744	0.07	0.936
	Skin - Not Sun Exposed (Suprapubic)	517	-0.0757	0.007	0.958
	Adipose - Subcutaneous	581	-0.078	1.20E-03	1
	Brain - Cerebellar Hemisphere	175	-0.0793	0.3	0.912
	Uterus	129	-0.0808	0.1	0.943
	Nerve - Tibial	532	-0.0826	7.40E-05	1
	Cells - EBV-transformed lymphocytes	147	-0.0828	0.09	0.931
	Stomach	324	-0.0844	0.02	0.995
	Esophagus - Muscularis	465	-0.0876	4.80E-04	1
	Esophagus - Gastroesophageal Junction	330	-0.0936	4.50E-03	1
	Ovary	167	-0.0942	0.02	0.981
	Cells - Cultured fibroblasts	483	-0.098	4.10E-07	1
	Heart - Left Ventricle	386	-0.0992	4.00E-04	0.996
	Thyroid	574	-0.0994	1.30E-06	1
	Adrenal Gland	233	-0.12	1.00E-02	0.998
	Artery - Aorta	387	-0.126	1.10E-05	1
	Pancreas	305	-0.126	0.007	0.995
	Vagina	141	-0.127	4.80E-03	0.992
	Artery - Coronary	213	-0.132	2.30E-04	1
	Testis	322	-0.158	1.70E-03	0.985
	Pituitary	237	-0.16	1.70E-03	1
	Brain - Substantia nigra	114	-0.17	0.02	0.98
	Brain - Cerebellum	209	-0.189	0.01	0.971
	Brain - Nucleus accumbens (basal ganglia)	202	-0.21	2.40E-05	1
	Brain - Hippocampus	165	-0.224	7.60E-04	1
	Brain Putamen (basal ganglia)	170	-0.231	4.90E-04	1
	Brain-Caudate (basal ganglia)	194	-0.231	1.50E-05	1
	Brain - Frontal Cortex (BA9)	175	-0.237	1.30E-05	1
	Brain - Cortex	205	-0.276	8.70E-06	0.983
	Brain - Anterior cingulate cortex (BA24)	147	-0.28	9.60E-05	0.999
	Brain - Amygdala	129	-0.313	2.10E-08	1
	Brain - Hypothalamus	170	-0.316	1.00E-07	1
	Brain - Spinal cord (cervical c-1)	126	-0.405	2.00E-08	1

Gene	Tissue	Samples	NES	p-value	m-value
UBE2Z	Small Intestine - Terminal Ileum	174	0.147	0.03	0.16
	Cells-EBV-transformed lymphocytes	147	0.146	0.06	0.152
	Whole Blood	670	0.114	3.90E-05	0.331
	Liver	208	0.0831	0.09	0.154
	Colon - Transverse	368	0.0738	0.03	0.189
	Cells - Cultured fibroblasts	483	0.0638	0.03	0.215
	Artery - Coronary	213	0.0548	0.2	0.159
	Pancreas	305	0.0533	0.2	0.108
	Spleen	227	0.0483	0.5	0.142
	Heart - Left Ventricle	386	0.0463	0.08	0.177
	Adipose - Subcutaneous	581	0.0459	0.1	0.155
	Lung	515	0.0452	0.09	0.191
	Esophagus - Gastroesophageal Junction	330	0.0398	0.3	0.145
	Esophagus - Mucosa	497	0.0355	0.2	0.133
	Prostate	221	0.0332	0.6	0.146
	Colon - Sigmoid	318	0.0313	0.4	0.167
	Adipose - Visceral (Omentum)	469	0.0286	0.3	0.093
	Testis	322	0.024	0.2	0.123
	Stomach	324	0.024	0.5	0.119
	Ovary	167	0.0237	0.6	0.167
	Artery - Aorta	387	0.0214	0.5	0.093
	Breast - Mammary Tissue	396	0.019	0.6	0.071
	Esophagus Muscularis	465	0.00997	0.7	0.051
	Brain - Cerebellar Hemisphere	175	0.00877	0.9	0.104
	Nerve - Tibial	532	0.00852	0.8	0.088
	Thyroid	574	0.00794	0.8	0.033
	Kidney - Cortex	73	0.00501	0.9	0.157
	Heart - Atrial Appendage	372	-0.00293	0.9	0.06
	Brain - Cerebellum	209	-0.00469	0.9	0.153
	Brain - Cortex	205	-0.00588	0.9	0.144
	Adrenal Gland	233	-0.0244	0.6	0.208
	Artery - Tibial	584	-0.0287	0.3	0.09
	Brain - Nucleus accumbens (basal ganglia)	202	-0.0319	0.5	0.192
	Brain Frontal Cortex (BA9)	175	-0.0436	0.3	0.264
	Muscle - Skeletal	706	-0.0477	0.05	0.244
	Pituitary	237	-0.0494	0.3	0.218
	Brain - Anterior cingulate cortex (BA24)	147	-0.058	0.3	0.221
	Skin - Sun Exposed (Lower leg)	605	-0.0587	0.01	0.408
	Uterus	129	-0.061	0.3	0.213
	Vagina	141	-0.0732	0.3	0.251
	Skin - Not Sun Exposed (Suprapubic)	517	-0.0908	1.10E-03	0.625
	Brain - Hypothalamus	170	-0.0951	0.07	0.345
	Brain-Hippocampus	165	-0.0976	0.06	0.467
	Minor Salivary Gland	144	-0.0982	0.2	0.259
	Brain Caudate (basal ganglia)	194	-0.0989	0.1	0.249
	Brain - Putamen (basal ganglia)	170	-0.163	0.02	0.453
	Brain-Spinal cord (cervical c-1)	126	-0.233	2.90E-05	0.655
	Brain - Substantia nigra	114	-0.258	2.60E-03	0.469
	Brain - Amygdala	129	-0.273	5.30E-04	0.645

Gene	Tissue	Samples	NES	p-value	m-value
IGF2BP1	Artery - Tibial	584	0.0302	0.6	0.017
	Cells - Cultured fibroblasts	483	0.01	0.8	0.009
	Thyroid	574	-	-	-
	Small Intestine - Terminal Ileum	174	-	-	-
	Brain - Frontal Cortex (BA9)	175	-	-	-
	Skin - Not Sun Exposed (Suprapubic)	517	-	-	-
	Vagina	141	-	-	-
	Whole Blood	670	-	-	-
	Breast - Mammary Tissue	396	-	-	-
	Pituitary	237	-	-	-
	Minor Salivary Gland	144	-	-	-
	Adipose - Subcutaneous	581	-	-	-
	Adrenal Gland	233	-	-	-
	Heart - Atrial Appendage	372	-	-	-
	Brain - Amygdala	129	-	-	-
	Stomach	324	-	-	-
	Brain - Caudate (basal ganglia)	194	-	-	-
	Colon - Transverse	368	-	-	-
	Brain - Cerebellum	209	-	-	-
	Esophagus - Muscularis	465	-	-	-
	Liver	208	-	-	-
	Brain - Hypothalamus	170	-	-	-
	Prostate	221	-	-	-
	Pancreas	305	-	-	-
	Nerve - Tibial	532	-	-	-
	Spleen	227	-	-	-
	Colon - Sigmoid	318	-	-	-
	Brain - Anterior cingulate cortex (BA24)	147	-	-	-
	Muscle - Skeletal	706	-	-	-
	Brain - Hippocampus	165	-	-	-
	Brain-Cortex	205	-	-	-
	Heart - Left Ventricle	386	-	-	-
	Uterus	129	-	-	-
	Ovary	167	-	-	-
	Esophagus - Gastroesophageal Junction	330	-	-	-
	Brain - Spinal cord (cervical c-1)	126	-	-	-
	Adipose - Visceral (Omentum)	469	-	-	-
	Brain - Nucleus accumbens (basal ganglia)	202	-	-	-
	Brain - Cerebellar Hemisphere	175	-	-	-
	Esophagus - Mucosa	497	-	-	-
	Artery - Aorta	387	-	-	-
	Brain - Putamen (basal ganglia)	170	-	-	-
	Lung	515	-	-	-
	Brain - Substantia nigra	114	-	-	-
	Skin - Sun Exposed (Lower leg)	605	-	-	-
	Cells - EBV-transformed lymphocytes	147	-0.0388	0.8	0.352
	Artery - Coronary	213	-0.0485	0.5	0.277
	Kidney - Cortex	73	-0.137	0.08	0.715
	Testis	322	-0.18	9.00E-07	1

Disease Models & Mechanisms • Supplementary information

Table S2. Sleep and cardiac parameters of 1-week-old male flies with neuronal (Elav-Gal4, N=16-24) or cardiac (Hand-Gal4, N=30-34) KD of CVD- and insomnia-related genes compared with their respective UAS controls.

	Total Sleep			Day Sleep			Night Sleep			Total Activity		
	Mean	SEM	p-value	Mean	SEM	p-value	Mean	SEM	p-value	Mean	SEM	p-value
ATPSynC UAS/+	895.6	35.43		370.6	21.46		524.9	21.26		1515	119.5	
Elav>ATPSynC	1092	23.22	0.0001	460.1	14.93	0.0042	632.1	12.24	0.0012	483.2	34.29	<0.0001
Bruce UAS/+	1045	16.74		472.6	13.75		572.8	7.19		848.1	35.82	
Elav>Bruce	781.3	38.81	<0.0001	335.5	20.99	<0.0001	445.8	26.39	<0.0001	1403	99.87	<0.0001
Lsn UAS/+	1082	12.83		482.7	9.595		599.4	8.149		817.3	32.39	
Elav>Lsn	968.4	35.02	0.0476	391.5	21.92	0.0023	576.9	17.22	0.8892	1087	120	0.1317
Imp UAS/+	899.2	26.23		474.8	15.53		424.4	23.01		1396	84.32	
Elav>Imp	1047	40.47	0.0122	617.4	19.59	<0.0001	438.3	27.57	0.9862	943	104.1	0.0056

	Day Activity			Night Activity			Total Bout Number			Total Bout Length		
	Mean	SEM	p-value	Mean	SEM	p-value	Mean	SEM	p-value	Mean	SEM	p-value
ATPSynC UAS/+	976.2	75.4		538.8	61.3		32.16	1.174		29.07	2.027	
Elav>ATPSynC	309.1	20.33	<0.0001	174.1	21.62	<0.0001	28.88	1.572	0.3643	37.51	2.379	0.0458
Bruce UAS/+	500.4	27.27		347.7	22.21		30.38	1.059		35.49	1.692	
Elav>Bruce	706.5	50.41	0.0206	696.2	64.11	<0.0001	23.88	0.9378	0.0021	32.65	2.091	0.7955
Lsn UAS/+	495.9	25.38		321.3	23.12		23.15	1.319		49.54	2.717	
Elav>Lsn	693.5	67.13	0.0483	393.9	56.58	0.7554	24.42	0.8305	0.9464	37.38	1.388	0.001
Imp UAS/+	615.8	50.76		779.9	64.3		22.64	1.954		42.56	3.128	
Elav>Imp	328.5	77.01	0.004	614.4	42.32	0.1187	36.8	2.132	<0.0001	27.98	2.34	0.0003

	HP			AI			DI			DD			SD			FS		
	Mean	SEM	p-value	Mean	SEM	p-value	Mean	SEM	p-value	Mean	SEM	p-value	Mean	SEM	p-value	Mean	SEM	p-value
ATPSynC UAS/+	0.5455	0.0283		0.1437	0.04035		0.2736	0.02262		70.51	1.128		37.26	1.615		0.4744	0.01597	
Hand>ATPSynC	0.977	0.04622	<0.0001	0.497	0.1101	<0.0001	0.7276	0.0446	<0.0001	64.45	2.184	0.0301	49.24	1.294	<0.0001	0.2263	0.01872	<0.0001
Bruce UAS/+	0.5572	0.02141		0.1028	0.01108		0.2959	0.0191		72.85	1.173		40.84	0.8314		0.4394	0.007475	
Hand>Bruce	0.6843	0.05062	0.0796	0.1316	0.02874	0.9792	0.4493	0.04611	0.0066	74.07	0.9056	0.9657	42.35	0.7595	0.9036	0.4274	0.009609	0.9396
Lsn UAS/+	0.6254	0.03659		0.08183	0.01714		0.3628	0.03425		68.83	1.506		41.14	0.7055		0.3981	0.01059	
Hand>Lsn	0.5042	0.0323	0.0485	0.04489	0.007698	0.9599	0.2966	0.02676	0.5908	95.71	2.469	<0.0001	79.06	2.858	<0.0001	0.1782	0.01695	<0.0001
Imp UAS/+	0.6683	0.05136		0.08736	0.0141		0.3719	0.04294		69.6	1.44		41.31	1.013		0.4052	0.01024	
Hand>Imp	0.4827	0.03134	0.0029	0.1107	0.01835	0.9904	0.2669	0.0277	0.1124	67.94	1.235	0.8974	40.13	1.197	0.9576	0.4102	0.01289	0.9977

Table S3. Circadian rhythmicity of flies with neuronal-specific KD of CVD- and insomnia-related genes.

Genotype	N Flies	Percent Rhythmic	Rhythmic	Arrhythmic	Rhytmic Only					
					Avg FFT	St. Err.	Adj. P-value	Avg Period	St. Err.	Adj. P-value
Elav>w1118	14	92.85714286	13	1	0.00752308	0.0009368	-	24.0269231	0.1655	-
Elav>ATPSynC	17	41.17647059	7	10	0.00394286	0.000268	0.052	24.43	0.768	0.7903
Elav>Bruce	23	100	23	0	0.00972174	0.0007852	0.1278	24.2747826	0.2109	0.8646
Elav>Lsn	13	92.30769231	12	1	0.00705	0.0006814	0.9667	24.1866667	0.3199	0.9713
Elav>Imp	18	0	0	18	-	-	-	-	-	-

Table S4. Summary table of non-cell-autonomous phenotypes observed upon KD of CVD- and insomnia-related genes.

	ATPsynC	Bruce	Lsn	Imp
Total Sleep	+	none	none	+
Daytime Sleep	none	-	none	+
Nighttime Sleep	+	none	+	none
Activity	-	none	none	-
Total Bout Number	+	none	none	+
Total Bout Length	none	none	none	-
Heart Period	+	none	-	-
Arrhythmia Index	+	none	none	none
Diastolic Interval	+	none	-	-
Diastolic Diameter	-	none	+	none
Systolic Diameter	+	none	+	none
Fractional Shortening	-	none	-	none

Disease Models & Mechanisms • Supplementary information

Table S5. Genotypes of RNAi lines.

Line	Genotype	Construct ID
V106834	P{KK108875}VIE-260B	KK108875
BL35464	y[1] sc[*] v[1] sev[21]; P{y[+t7.7] v[+t1.8]=TRiP.GL00390}attP2	GL00390
BL57705	y[1] sc[*] v[1] sev[21]; P{y[+t7.7] v[+t1.8]=TRiP.HMC04894}attP40	HMC04894
V110350	P{KK100908}VIE-260B	KK100908
V21658	w1118; P{GD10787}v21658	GD10787
BL38289	y[1] sc[*] v[1] sev[21]; P{y[+t7.7] v[+t1.8]=TRiP.HMS01747}attP40	HMS01747
V107620	P{KK100132}VIE-260B	KK100132
V48309	w1118; P{GD16949}v48309/CyO	GD16949
BL51814	y[1] sc[*] v[1] sev[21]; P{y[+t7.7] v[+t1.8]=TRiP.HMC03385}attP2/TM3, Sb[1] Ser[1]	HMC03385
V20321	w1118; P{GD9232}v20321	GD9232
V20322	w1118; P{GD9232}v20322/CyO	GD9232
BL38219	y[1] v[1]; P{y[+t7.7] v[+t1.8]=TRiP.GL00660}attP40	GL00660
BL55645	y[1] sc[*] v[1] sev[21]; P{y[+t7.7] v[+t1.8]=TRiP.HMC03794}attP40	HMC03794
BL34977	y[1] sc[*] v[1] sev[21]; P{y[+t7.7] v[+t1.8]=TRiP.HMS01168}attP2	HMS01168

Table S6. Sleep and cardiac parameters of 1-week-old male flies with neuronal (Elav>Gal4) or cadiac (Hand-Gal4) KD of CVD- and insomnia-related genes compared with their respective RNAi controls.

	Total Sleep	Day Sleep	Night Sleep	Total Activity	Day Activity	Night Activity	Total Bout Number	Total Bout Length
Elav-Gal4> (N=16-24)	p-value	p-value	p-value	p-value	p-value	p-value	p-value	p-value
Elav/+ vs. Bruce	0.6677	0.0074	>0.9999	0.9793	0.652	>0.9999	>0.9999	0.9441
Elav/+ vs. Lsn	0.2971	0.9083	<0.0001	0.7172	0.8517	0.001	>0.9999	0.9957
Elav/+ vs. Imp	0.0012	<0.0001	>0.9999	0.0073	0.0104	0.9425	<0.0001	0.0322
Elav/+ vs. ATPSynC	<0.0001	0.9617	<0.0001	<0.0001	0.0094	<0.0001	0.2283	0.9992
Elav/+ vs. VDRC CTL	0.9981	0.0065	<0.0001	0.9324	0.6163	0.0029	>0.9999	0.0034
Elav/+ vs. BDSC CTL	0.0025	<0.0001	0.2789	>0.9999	0.0032	0.0169	0.2204	0.5467
ATPSynC vs. BDSC CTL	<0.0001	<0.0001	0.0015	<0.0001	<0.0001	0.0206	>0.9999	>0.9999
Bruce vs. VDRC CTL	0.1962	>0.9999	<0.0001	0.28	>0.9999	0.0036	>0.9999	0.0053
Lsn vs. VDRC CTL	0.8243	0.3748	>0.9999	>0.9999	>0.9999	>0.9999	>0.9999	0.9988
Imp vs. VDRC CTL	0.0177	<0.0001	0.0001	0.2233	<0.0001	0.1571	<0.0001	0.0011

	HP	AI	DI	DD	SD	FS
Hand-Gal4> (N=30-33)	p-value	p-value	p-value	p-value	p-value	p-value
Hand/+ vs. ATPSynC	0.0008	<0.0001	0.0001	0.073	0.0005	<0.0001
Hand/+ vs. Bruce	>0.9999	0.9997	>0.9999	0.7041	0.9895	>0.9999
Hand/+ vs. Lsn	0.0377	0.9998	0.1586	<0.0001	<0.0001	<0.0001
Hand/+ vs. Imp	0.0126	>0.9999	0.0399	0.9277	>0.9999	0.9972
Hand/+ vs. VDRC CTL	0.0035	>0.9999	0.0156	0.6353	0.3112	0.6292
Hand/+ vs. BDSC CTL	0.9992	0.0011	0.9989	0.5435	0.3704	0.9104
ATPSynC vs. VDRC CTL	<0.0001	<0.0001	<0.0001	0.9685	<0.0001	<0.0001
Bruce vs. VDRC CTL	0.0067	0.9972	0.0096	0.0115	0.0249	0.7187
Lsn vs. VDRC CTL	0.9985	>0.9999	0.996	<0.0001	<0.0001	<0.0001
Imp vs. VDRC CTL	>0.9999	>0.9999	>0.9999	>0.9999	0.4381	0.1383
